# Difference of binding modes among three ligands to a receptor mSin3B corresponding to their inhibitory activities

**DOI:** 10.1038/s41598-021-85612-9

**Published:** 2021-03-17

**Authors:** Tomonori Hayami, Narutoshi Kamiya, Kota Kasahara, Takeshi Kawabata, Jun-ichi Kurita, Yoshifumi Fukunishi, Yoshifumi Nishimura, Haruki Nakamura, Junichi Higo

**Affiliations:** 1 Institute for Protein Research, Osaka University, 3-2 Yamada-oka, Suita, Osaka 565-0871 Japan; 2 Graduate School of Simulation Studies, University of Hyogo, 7-1-28 Minatojima Minamimachi, Chuo-ku, Kobe, Hyogo 650-0047 Japan; 3 College of Life Sciences, Ritsumeikan University, 1-1-1 Noji-higashi, Kusatsu, Shiga 525-8577 Japan; 4 Graduate School of Medical Life Science, Yokohama City University, 1-7-29 Suehiro-cho, Tsurumi-ku, Yokohama, 230-0045 Japan; 5 Cellular and Molecular Biotechnology Research Institute, National Institute of Advanced Industrial Science and Technology (AIST), 2-3-26, Aomi, Koto-ku, Tokyo 135-0064 Japan; 6 Graduate School of Integrated Sciences for Life, Hiroshima University, 1-4-4 Kagamiyama, Higashi-Hiroshima, 739-8258 Japan

**Keywords:** Computational biophysics, Protein function predictions

## Abstract

A preceding experiment suggested that a compound, which inhibits binding of the REST/NRSF segment to the cleft of a receptor protein mSin3B, can be a potential drug candidate to ameliorate many neuropathies. We have recently developed an enhanced conformational sampling method, genetic-algorithm-guided multi-dimensional virtual-system-coupled canonical molecular dynamics, and in the present study, applied it to three systems consisting of mSin3B and one of three compounds, sertraline, YN3, and acitretin. Other preceding experiments showed that only sertraline inhibits the binding of REST/NRSF to mSin3B. The current simulation study produced the spatial distribution of the compounds around mSin3B, and showed that sertraline and YN3 bound to the cleft of mSin3B with a high propensity, although acitretin did not. Further analyses of the simulation data indicated that only the sertraline–mSin3B complex produced a hydrophobic core similar to that observed in the molecular interface of the REST/NRSF-mSin3B complex: An aromatic ring of sertraline sunk deeply in the mSin3B’s cleft forming a hydrophobic core contacting to hydrophobic amino-acid residues located at the bottom of the cleft. The present study proposes a step to design a compound that inhibits competitively the binding of a ligand to its receptor.

## Introduction

Neural restrictive silencer factor (NRSF), which is also known as Repressor-element 1 silencing transcription factor (REST)^1,2^, is a fundamental repressor, which binds to repressor-element 1 (re1) or neural restrictive silencer element (nrse) of many neuronal genes^3,4^. Importantly, overexpression of REST/NRSF or dysregulation of its cellular expression pattern is related to many neuropathies: Medulloblastoma^5,6^, malignant pediatric brain tumor^7^, glioblastoma^8,9^, Huntington’s disease^10–13^, neuropathic pain^14,15^, Parkinson’s disease^16^, autism^17^, and fibromyalgia^18^.

REST/NRSF mediates transcriptional repression recruiting two corepressor complexes: REST/NRSF binds to mSin3 at its N-terminus and to CoREST plus the histone H3K9 methyltransferase G9a at its C-terminus^19^. The mSin3 complex, which contains two histone deacetylases HDAC1 and HDAC2^20^, was implicated as an important epigenetic regulator in cancer^21^. The corepressor mSin3B, an isoform of mSin3, consists of four paired amphipathic helix domains (PAH1–PAH4) connected by linkers among the domains, and an intrinsically disordered region of REST/NRSF binds to the cleft of PAH1 of mSin3B^22^.

Interestingly, an NMR experiment has shown that the disordered regions of REST/NRSF folds into a helix when binding to the hydrophobic cleft of the PAH1 domain^22^ (coupled folding and binding^23–25^). See Supplementary Figure S1 for the PAH1 structure and the cleft position in the domain. A microscopic mechanism for the coupled folding and binding of this system was elucidated by our earlier computational study^26^. The NMR work provided a useful strategy for drug discovery: A compound that inhibits the binding of REST/NRSF to the PAH1 cleft of mSin3B can be a potential drug candidate to ameliorate the neuropathies^18,27–30^, and many compounds have been examined^18,22,31^.

In fact, Ueda et al. analyzed the NMR complex structure of REST/NRST and the PAH1 domain of mSin3B, and proposed a compound mS-11 to inhibit the binding of REST/NRSF to the PAH1 domain^18^. This compound mimics the helical structure of a four-residue segment (Leu46-Ile47-Met48-Leu49) of REST/NRSF in the bound form, and importantly, this compound inhibited actually the binding of REST/NRST to the PAH1 domain. We call this four-residue segment a LIML sequence in this study.

Recently, we developed a generalized ensemble method, multi-dimensional virtual-system coupled molecular dynamics (mD-VcMD) simulation^32,33^. This method enhances conformational sampling of biomolecules in an explicit solvent: By introducing multiple reaction coordinates (RCs) in the molecular system, the conformational motions of the molecules are enhanced with controlling the values of the multiple RCs. The search region in the RC space is expanded through iterative simulation. Importantly, a thermodynamic weight is assigned to each of the sampled conformations, and various thermodynamic quantities of the system are computed from the weighted snapshots. Then, we extended mD-VcMD by using a genetic algorithm and named the method as a genetic-algorithm-guided mD-VcMD (GA-guided mD-VcMD) simulation^34,35^ where the genetic algorithm supports expansion of the search range effectively. We have shown that the sampling efficiency of the GA-guided mD-VcMD is significantly higher than that of the original mD-VcMD^34^.

In the present study, we investigate the spatial distribution of three compounds sertraline, YN3, and acitretin, respectively, around the PAH1 domain of mSin3B, which are obtained from the GA-guided mD-VcMD simulation of the system. The chemical structures of the three compounds are shown in Supplementary Figure S2. We note that preceding experiments on sertraline and YN3 have shown that only sertraline exhibited medulloblastoma cell growth inhibitory activity, although both compounds bound to the PAH1 domain of mSin3B^31^. Another preceding experiment has shown that no inhibitory activities were detected for acitretin whereas it also binds to the PAH1 domain (personal communication with Nishimura). We show that the spatial distribution of the three compounds from the simulations rationally explains why only sertraline exhibited the inhibitory activity. Based on these computational results, we discuss a strategy to develop a drug candidate.

## Materials and methods

In this study, we denote the PAH1 domain of mSin3B simply as “mSin3B” for convenience. Besides, a system composed of sertraline and mSin3B is referred to as a “sertraline–mSin3B” system even when the two molecules are apart to each other during the simulation. Similarly, a system of YN3 and mSin3B is done to as a “YN3–mSin3B” system, and that of acitretin and mSin3B as a “acitretin–mSin3B” system.

### Three reaction coordinates

First, we introduced multiple RCs in the system, where an RC was defined by the distance between centers of mass of two atom groups. Supplementary Section 1 presents a detailed method to set an RC in the system, and Supplementary Figure S3 shows schematically the RC, which is set in a system.

For each the of ligand–receptor systems, we introduced three RCs, denoted as $\lambda^{\left( h \right)}$ ($h = \alpha ,\;\beta ,\;\gamma$), presented in Supplementary Figure S2. Schematic representation for the three RCs is given in Supplementary Figure S4a. We briefly explain here the RCs as follows: The two atom groups $G_{\alpha }^{A}$ (red-colored segments in Supplementary Figure S2) and $G_{\alpha }^{B}$ (blue-colored segments) define the first RC $\lambda^{\left( \alpha \right)}$. We can imagine readily that the move of $\lambda^{\left( \alpha \right)}$ opens/closes the cleft. Atom groups $G_{\beta }^{A}$ and $G_{\gamma }^{A}$ are respectively green-colored and cyan-colored segments in mSin3B, and $G_{\beta }^{B}$ and $G_{\gamma }^{B}$ are purple-colored and orange-colored portions in the ligand (Supplementary Figure S2). The moves of $\lambda^{\left( \beta \right)}$ (distance between $G_{\beta }^{A}$ and $G_{\beta }^{B}$) and $\lambda^{\left( \gamma \right)}$ (distance between $G_{\gamma }^{A}$ and $G_{\gamma }^{B}$) control the ligand approaching/departing to mSin3B. When $\lambda^{\left( \beta \right)}$ increases with decreasing $\lambda^{\left( \gamma \right)}$ or when $\lambda^{\left( \beta \right)}$ decreases with increasing $\lambda^{\left( \gamma \right)}$, the ligand rotates.

We note that the selection of RCs can be arbitrary in theory if a very long simulation is possible. However, the selection is essentially important to raise the efficiency in an actual simulation. Detailed information for the atom groups is given in Supplementary Table S1.

To study molecular binding extensively, the multi-dimensional RC region should involve both the unbound and bound conformations. For this purpose, we set the variable ranges for the three RCs wide enough (Supplementary Table S2). The term “multi-dimensional (mD)” means three-dimensional (3D) in this study, whereas the current method is applicable to any dimensional RC space.

### Initial conformations of simulation

After setting the RCs above, the initial conformation of simulation was generated. First, the tertiary structure of the receptor mSin3B (PAH1 domain) was taken from the PDBj site (https://pdbj.org/) (PDB ID: 2CZY), in which the receptor binds to the REST/NRSF fragment. After removing REST/NRSF from the complex, we introduced a ligand (sertraline, YN3, or acitretin) near mSin3B. As explained later, we randomized the position of the ligand to generate the initial conformations of the GA-guided mD-VcMD simulation, where the ligand was distant from the binding cleft of mSin3B.

Next, we put the two molecules (ligand and mSin3B) generated above in a periodic boundary box (size is 70.0 Å^3^) filled by water molecules, and removed water molecules that overlapped to mSin3B or the ligand. Then Na^+^ and Cl^−^ ions were introduced with randomly replacing water molecules by ions. The number of ions was set to a physiological ionic concentration with neutralizing the net charge of the whole system to zero. The resultant sertraline–mSin3B system consists of 33,543 atoms (1200 atoms for mSin3B, 38 atoms for sertraline, 10,749 water molecules, 29 Na^+^, 29 Cl^−^), the YN3–mSin3B system does of 33,533 atoms (1200 atoms for mSin3B, 40 atoms for YN3, 10,745 water molecules, 29 Na^+^, 29 Cl^−^), and the acitretin–mSin3B system does of 33,525 atoms (1200 atoms for mSin3B, 50 atoms for acitretin, 10,739 water molecules, 29 Na^+^, 29 Cl^−^). After a short energy minimization, a short constant-volume and constant-temperature (300 K) simulation (NVT simulation) was performed. Then, a constant-pressure (1 atm) and constant-temperature (300 K) simulation (NPT simulation) was performed to relaxed the box size. The resultant box size was 68.920^3^ Å^3^, 68.909^3^, and 68.909^3^ for the sertraline–mSin3B, YN3–mSin3B, and acitretin–mSin3B systems, respectively. Those computations were done by using a program package myPresto/psygene^36^. The force fields used for those simulations are described later.

Whereas the PAH1 domain is linked to the PAH2 domain by a long flexible linker in a real cell, only the PAH1 domain was computed in our simulation, and the inter-domain linker was treated as the C-terminal tail. This tail might be inserted into the binding cleft of PAH1 domain incidentally during the simulation. It is likely that the incidental insertion of the inter-domain linker into the cleft does not happen if the PAH1 and PAH domains are connected by the linker. Thus, to prevent this incidental and artificial event, we applied weak restraints to the C-terminal tail (see Supplementary Section 2). By the restraints, the C-terminal did not move to the binding cleft, and fluctuated around the initial conformation (NMR structure; PDB ID: 2CZY) during the simulation.

To generate the initial conformations of simulation, where the ligand is distant from the binding cleft of mSin3B, we applied interactions between mSin3B and the ligand so that the RCs fall in the following ranges: 15 Å < *λ*^(*α*)^ < 16 Å, 24 Å < *λ*^(*β*)^ < 25 Å, and 24 Å < *λ*^(*γ*)^ < 25 Å. Then, with applying these interactions we performed 256 runs starting from the last snapshot of the NPT simulation done above. Supplementary Figure S5 display some of the last conformations picked randomly from those 256 runs for the three systems. We used those 256 conformations for the initial conformation of GA-guided mD-VcMD. Apparently, the ligand in these conformations was distant from the cleft of mSin3B (i.e., REST/NRSF position). These figures also display the REST/NRSF fragment binding to the cleft of mSin3B (PDB ID: 2CZY), whereas REST/NRSF did not exist in the current simulation.

### The GA-guided mD-VcMD

Methodological details for GA-guided mD-VcMD are explained elsewhere^34^. Here, we explain the outline of GA-guided mD-VcMD and resultant quantities. This method controls the system’s motions by modulating the three RCs $\lambda^{\left( h \right)}$ ($h = \alpha ,\;\beta ,\;\gamma$). Supplementary Figure S4b presents schematically a distribution of the system’s conformation in the 3D RC space resulted from the moves of $\lambda^{\left( h \right)}$.

The outline of the method is as follows: First, the entire 3D RC space is divided into many small zones. Then, the GA-guided mD-VcMD simulation provides a conformational distribution function $Q_{cano} \left( {\lambda^{\left( \alpha \right)} ,\lambda^{\left( \beta \right)} ,\lambda^{\left( \gamma \right)} } \right)$ of the system, where $Q_{cano} \left( {\lambda^{\left( \alpha \right)} ,\lambda^{\left( \beta \right)} ,\lambda^{\left( \gamma \right)} } \right)$ is the probability of existence at position $\left[ {\lambda^{\left( \alpha \right)} ,\lambda^{\left( \beta \right)} ,\lambda^{\left( \gamma \right)} } \right]$ in the 3D RC space constructed by $\lambda^{\left( \alpha \right)}$, $\lambda^{\left( \beta \right)}$, and $\lambda^{\left( \gamma \right)}$. Because the conformational space of a biological molecular system is wide, the GA-guided mD-VcMD is executed via iterative simulations, during which the sampled RC region is expanded. The simulation is terminated when $Q_{cano} \left( {\lambda^{\left( \alpha \right)} ,\lambda^{\left( \beta \right)} ,\lambda^{\left( \gamma \right)} } \right)$ has converged. We discuss the convergence quantitatively later. After the convergence, a thermodynamic weight is assigned to each of stored snapshots using $Q_{cano} \left( {\lambda^{\left( \alpha \right)} ,\lambda^{\left( \beta \right)} ,\lambda^{\left( \gamma \right)} } \right)$, and the ensemble of the snapshots can be regarded as a thermally equilibrated conformational ensemble (canonical ensemble)^34^. If the GA-guided mD-VcMD simulation is done at temperature $T$, then the canonical ensemble at $T$ is obtained.

We performed 256 runs for an iteration in the present study to raise the sampling efficiency further. A simple integration of the 256 trajectories can be regarded as a long single trajectory^37,38^. When the *M*-th iteration is finished, we have snapshots stored from the 1st to *M*th iterations. The 256 initial conformations for the (*M* + 1)th iteration were selected from those stored snapshots so that the conformations distributed as even as possible in the 3D-RC space. On the other hand, when an RC region was sampled poorly, we prepared the initial conformations around the poorly sampled region^34^. Because we obtained the canonical ensemble at 300 K as a result of the GA-guided mD-VcMD, we calculated the distribution function of various quantities in equilibrium at 300 K.

### Simulations

The actual parameter values for the GA-guised mD-VcMD are presented in Supplementary Section 3. The simulation was performed using a computer program omegagene/myPresto^39^ with the following condition: SHAKE^40^ to fix the covalent-bond lengths related to hydrogen atoms, the Berendsen thermostat to control temperature^41^, the zero-dipole summation method^42–44^ to compute accurately and quickly the long-range electrostatic interactions, a time-step of 2 fs ($\Delta t = 2$ fs), and simulation temperature of 300 K. The Berendsen thermostat produces an ensemble that can approximate a canonical distribution for a many-atom system, whereas it generates a non-physical distribution for a small system^45^. To compute the original potential energy of the system, the Amber hybrid force fields (mixture parameter $w = 0.75$)^46^ was used for mSin3B, the TIP3P potential model for water molecules^47^, and the Joung–Cheatham model for chloride and sodium ions^48^.

The force fields for the sertraline, YN3, and acitretin were set as follows: First, the atomic partial charges were derived by quantum chemical calculations using Gaussian03^49^ at the HF/6-31G* level, followed by RESP fitting^50^. Then, those partial charges were incorporated into a GAFF (general amber force field) force-field file^51^. GAFF was designed to be compatible with conventional AMBER force-fields. The Amber hybrid force fields currently used for mSin3B was generated by ourselves with mixing Amber parm94^52^ and parm96^53^ force fields to treat both helical and stranded polypeptides^46^, and the difference between parm94 and parm96 exists only in the dihedral energy parameters. Therefore, the inter-molecular interaction energy between mSin3B and the ligands is invariant mechanically among the parm94, parm96, and hybrid force fields. Those force field parameters were used for the energy minimization, NVT, NPT, and the GA-guided mD-VcMD simulations.

### Spatial density of a compound around mSin3B

As mentioned above, the GA-guided VcMD simulation produces a conformational ensemble, where a thermodynamic weight at 300 K is assigned to each constituent conformation. Thus, we can calculate a spatial distribution function of any structural quantity from the ensemble. In this study, we compute the spatial density $\rho_{CG}^{\left( s \right)} \left( {\varvec{r}} \right)$, which is the probability of detecting the geometrical center (GC) of the ligand in the vicinity of a three-dimensional position ${\varvec{r}} = \left[ {{{x}},{{y}},{{z}}} \right]$ in the real space and the superscript $s$ is the system specifier: *s* = sertraline–mSin3B, YN3–mSin3B, or acitretin–mSin3B. The detailed computational procedure to calculate $\rho_{CG}^{\left( s \right)} \left( {\varvec{r}} \right)$ is explained in Supplementary Section 4. Other spatial density functions were calculated with the same manner: See Supplementary Eq. S4.

## Results

### Distribution of the system’s conformation in 3D RC space

We repeated 14, 13, and 27 iterations for the sertraline–mSin3B, YN3–mSin3B, and acitretin–mSin3B systems, respectively. For all of the three systems, an iteration was composed of 256 runs. Each run was performed for $3 \times 10^{6}$ steps (6 ns; time step $\Delta t = 2$ fs). Thus, the total simulation length was 21.504 µs (= 14 × 256 × 6 ns), 19.968 µs (= 13 × 256 × 6 ns), and 41.472 µs (= 27 × 256 × 6 ns) for the sertraline–mSin3B, YN3–mSin3B, and acitretin–mSin3B systems, respectively. A snapshot was stored every $1 \times 10^{5}$ steps (200 ps), yielding 107,520, 99,840, and 207,360 snapshots, for the sertraline–mSin3B, YN3–mSin3B, and acitretin–mSin3B systems, respectively.

**Figure 1:**
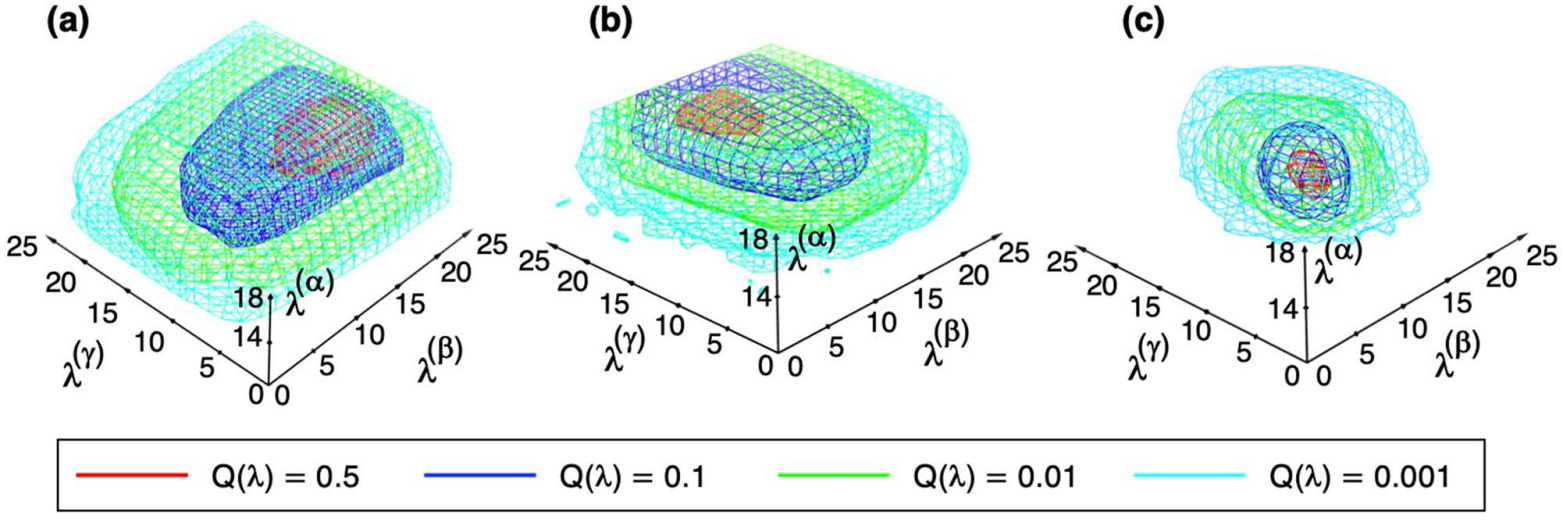
Density $Q_{cano} \left( {\lambda^{\left( \alpha \right)} ,\lambda^{\left( \beta \right)} ,\lambda^{\left( \gamma \right)} } \right)$ of (**a**) the sertraline–mSin3B, (**b**) YN3–mSin3B, and (**c**) acitretin–mSin3B systems in the 3D-RC space. In GA-guided mD-VcMD, the distribution is defined originally by $Q_{cano} \left( {L^{\left( \alpha \right)} , L^{\left( \beta \right)} ,L^{\left( \gamma \right)} } \right)$, where $L^{\left( \alpha \right)} ,\; L^{\left( \beta \right)}$, and $L^{\left( \gamma \right)}$ are respectively indices to specify the positions $\lambda^{\left( \alpha \right)}$, $\lambda^{\left( \beta \right)}$, and $\lambda^{\left( \gamma \right)}$ in the 3D-RC space. Then, we convert $\left[ {L_{i}^{\left( \alpha \right)} , L_{j}^{\left( \beta \right)} ,L_{k}^{\left( \gamma \right)} } \right]$ to: $\lambda_{i}^{\left( h \right)} = 0.5\left\{ {\left[ {\lambda_{i}^{\left( h \right)} } \right]_{min} + \left[ {\lambda_{i}^{\left( h \right)} } \right]_{max} } \right\}$, where $i = 1, \ldots ,n_{vs} \left( h \right)$ ($h = \alpha ,\;\beta ,\; \gamma$). See Supplementary Table S3 for values of $\left[ {\lambda_{i}^{\left( h \right)} } \right]_{min}$, $\left[ {\lambda_{i}^{\left( h \right)} } \right]_{max}$, and $n_{vs} \left( h \right)$. Then, $Q_{cano} \left( {\lambda^{\left( \alpha \right)} ,\lambda^{\left( \beta \right)} ,\lambda^{\left( \gamma \right)} } \right)$ is normalized so that the highest density is set to 1. Contour levels are presented by colors in inset.

Figure 1 demonstrates the conformational distributions of the three systems in the 3D RC space, where the density is normalized so that the largest density is set to 1.0. We checked the convergence of distribution during the iterations. The method to check the convergence is given in Supplementary Section 5. Supplementary Figures S6–S8 indicate that the regions with $Q_{cano} \left( {\lambda^{\left( \alpha \right)} ,\lambda^{\left( \beta \right)} ,\lambda^{\left( \gamma \right)} } \right) > 0.001$ are converged well. Although Fig. 1 is basically important to show that the sampling covered a wide conformational space, this figure is not useful for understanding the ligand’s distribution around mSin3B. In the next section, we analyze the ligand’s distribution using the canonical conformational ensemble.

### Distribution of ligands around mSin3B

**Figure 2:**
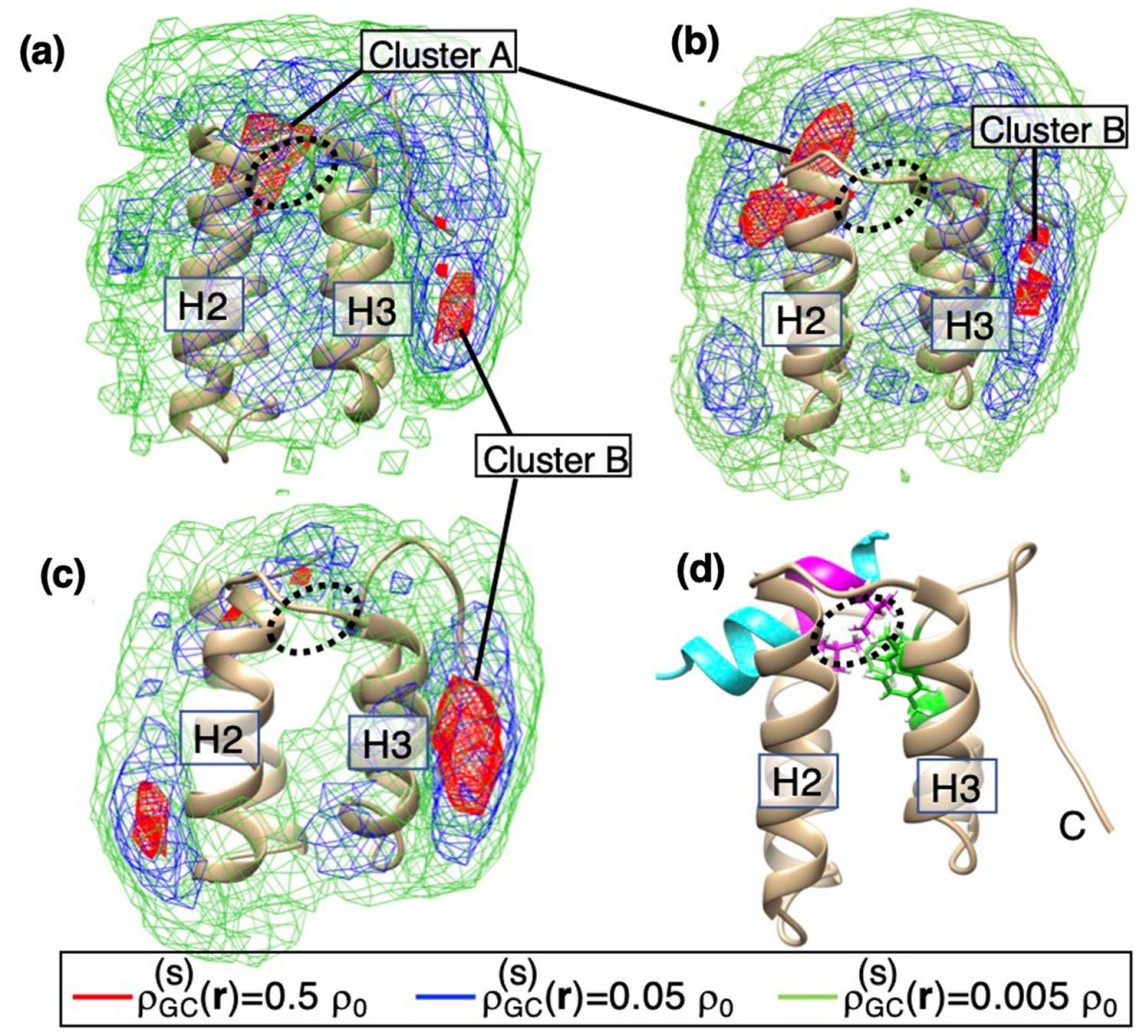
Spatial density $\rho_{CG}^{\left( s \right)} \left( {\varvec{r}} \right)$ (*s* = sertraline, YN3, or acitretin) of the geometric center (GC) at position ${\varvec{r}}$ for (**a**) the sertraline–mSin3B, (**b**) YN3–mSin3B, and (**c**) acitretin–mSin3B systems in the 3D real space. See Supplementary Section 4 for procedure to calculate $\rho_{CG}^{\left( s \right)} \left( {\varvec{r}} \right)$. Contour levels are shown in inset where $\rho_{0} = 0.001$. Displayed structure of mSin3B is that after NPT simulation for each system, where labels H2 and H3 represent helix 2 and helix 3 of mSi3B, respectively. The high-density cluster ($\rho_{CG}^{\left( s \right)} \left( {\varvec{r}} \right) > 0.5\rho_{0}$) in the cleft of mSin3B (PAH1) is named as Cluster A, and one near the N-terminal of mSin3B as Cluster B. (**d**) NMR structure of REST/NRSF-mSin3B complex (PDB ID: 2CZY), where cyan-colored model is REST/NRSF, and the magenta-colored segment is the LIML sequence of REST/NRSF. Label C indicates the position of the C-terminal tail of mSin3B. Two magenta-colored side-chains are Leu 46 and Leu 49 of the LIML sequence. Black broken-line circle indicates the position of the two sidechains. The circles in panels (**a**), (**b**), and (**c**) are presented to indicate the sidechain position of Leu 46 and Leu 49. Green-colored sidechains are Val 75, Phe 93, and Phe 96 of mSin3B (see also green-colored sidechains of Supplementary Figure S9a), which form a hydrophobic core with Leu 46 and Leu 49 of the LIML sequence.

We computed the spatial density $\rho_{CG}^{\left( s \right)} \left( {\varvec{r}} \right)$ for the geometric center of the ligands (Fig. 2). At the low-contour level ($\rho_{CG}^{\left( s \right)} \left( {\varvec{r}} \right) = 0.005\rho_{0}$); green-colored contours), the ligands distributed almost everywhere around mSin3B for all systems. This result is natural indicating that the sampling was done widely. With increasing the density level, the ligands tended to be localized at some surface regions of mSin3B ($\rho_{CG}^{\left( s \right)} \left( {\varvec{r}} \right) = 0.05\rho_{0}$; blue-colored contours). At the high-density level ($\rho_{CG}^{\left( s \right)} \left( {\varvec{r}} \right) = 0.5\rho_{0}$; red-colored contours), a high-density conformational cluster (labeled Cluster A in the figure) can be identified in the cleft of mSin3B for sertraline and YN3, whereas acitretin did not show a remarkable cluster in the cleft. This indicates that the ligand–mSin3B binding for sertraline and YN3 is stronger than that for acitretin.

Figure 2d displays the REST/NRSF-mSin3B complex structure solved by the NMR experiment^22^ from the same orientation of Fig. 2a–c. A view of the REST/NRSF-mSin3B complex from a different orientation is presented in Supplementary Figure S9a. The binding cleft of mSin3B is hydrophobic (Supplementary Figure S9b), and two residues Leu 46 and Leu 49 of the LIML sequence bind to the cleft forming a hydrophobic core with Val 75, Phe 93, and Phe 96 of mSin3B. See “Introduction” section for the LIML sequence. Interestingly, only the geometrical center of sertraline overlaps the position of Leu 46 and Leu 49 of the LIML sequence (black broken-line circle) in the REST/NRSF-mSin3B complex, when the spatial density is viewed at the contour level of $\rho_{CG}^{\left( s \right)} \left( {\varvec{r}} \right) = 0.5\rho_{0}$. Therefore, sertraline forms a tighter hydrophobic core with the binding cleft of mSin3B than the other ligands do.

We also observed a high-density cluster (Cluster B in Fig. 2) near the C-terminal tail of mSin3B. Because the C-terminal tail was restrained (see the Materials–and–Methods and Supplementary Section 2), the C-terminal tail did not fluctuate largely in the simulation in spite that the C-terminal tail is exposed to solvent. Therefore, it is likely that Cluster B was induced by this less-fluctuating C-terminal tail: Cluster B is an artificial cluster. Supplementary Figure S10 displays a sertraline’s conformation taken from Cluster B. The nitrogen atom of Ring B of sertraline interacts electrostatically to an oxygen atom of Asp 104 of the C-terminal tail. Ring A of sertraline interacts with the hydrophobic residue Ile 61 of helix H4 of mSin3B, and Ring C of sertraline does with the hydrophobic residue Val 62 of helix H4. Majority of conformations from Cluster B showed those interaction patterns. Therefore, it is likely that Cluster B disappears when the C-terminal tail is highly exposed to solvent and fluctuates largely.

### Radial distribution function of ligand-cleft distance

Figure 2 is insightful to guess the ligand–mSin3B interaction. Nevertheless,$\rho_{CG}^{\left( s \right)} \left( {\varvec{r}} \right)$ was calculated using the geometrical centers of the ligands. There is a possibility that some parts of the ligands contacted to the hydrophobic cleft tightly even when the geometrical center was distant from the cleft. For instance, acitretin is a long molecule, and then the head or tail of acitretin (Supplementary Figure S2c) may be inserted to the cleft keeping the geometrical center out of the cleft.

To make clear this point, we calculated a radial distribution function (RDF) between the ligand and three residues Val 75, Phe 93, and Phe 96 located in the mSin3B cleft. The computation procedure for RDF is given in Supplementary Section 6. First, we define the minimum heavy-atomic distance between the ligand and one of the three residues: $r_{R}^{\left( s \right)}$ where the subscript $R$ is the residue specifier (*R* = Val 75, Phe 93, or Phe 96) and the superscript $s$ is the system specifier defined before (*s* = sertraline, YN3, or acitretin). Then, the RDF $p\left( {r_{R}^{\left( s \right)} } \right)$ was calculated for each of the three distances.

**Figure 3:**
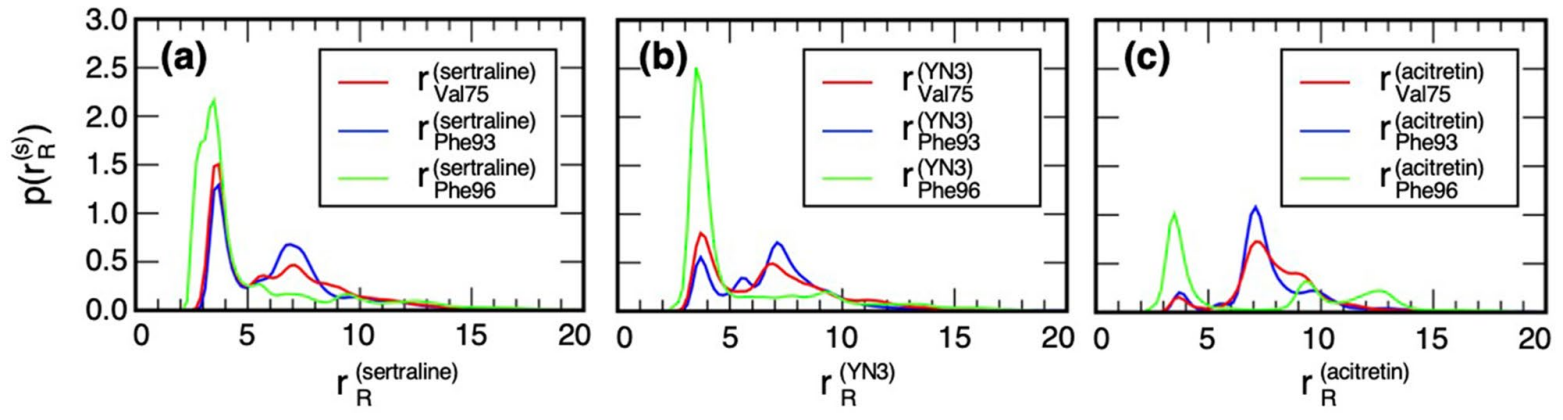
Radial distribution functions (RDFs) $p\left( {r_{R}^{\left( s \right)} } \right)$ for (**a**) the sertraline–mSin3B (*s* = sertraline), (**b**) YN3–mSin3B (*s* = YN3), and (**c**) acitretin–mSin3B systems (*s* = acitretin). Three RDFs $p\left( {r_{Val75}^{\left( s \right)} } \right)$, $p\left( {r_{Phe93}^{\left( s \right)} } \right)$, and $p\left( {r_{Phe96}^{\left( s \right)} } \right)$ are shown by different colors as indicated in insets of panels. See Supplementary Section 6 for procedure to calculate RDF.

Figure 3 demonstrates the resultant RDFs. For all of the systems, the function $p\left( {r_{Phe96}^{\left( s \right)} } \right)$ exhibited a peak at 3.5 Å regardless of the peak height. This result is plausible because the residue Phe 96 is located at the entrance of the cleft (Supplementary Figure S9a): The ligand can contact to Phe 96 without sinking into the cleft. More important RDFs are $p\left( {r_{Val75}^{\left( s \right)} } \right)$ and $p\left( {r_{Phe93}^{\left( s \right)} } \right)$ because Val 75 and Phe 93 are located at the bottom of the cleft. Apparently, the peaks of the RDFs at $r_{R}^{\left( s \right)}$ ~ 4 Å for the acitretin–mSin3B system were considerably smaller than those for sertraline–mSin3B and YN3–mSin3B systems. This means that acitretin did not interact frequently or tightly with the bottom of the cleft. The highest peaks were from the sertraline–mSin3B system (Fig. 3a), and the peaks from the YN3–mSin3B were intermediate between sertraline and acitretin (Fig. 3b). These results suggest that sertraline may resemble the ligand–receptor interaction mode found in the REST/NRSF-mSin3B complex.

**Figure 4:**
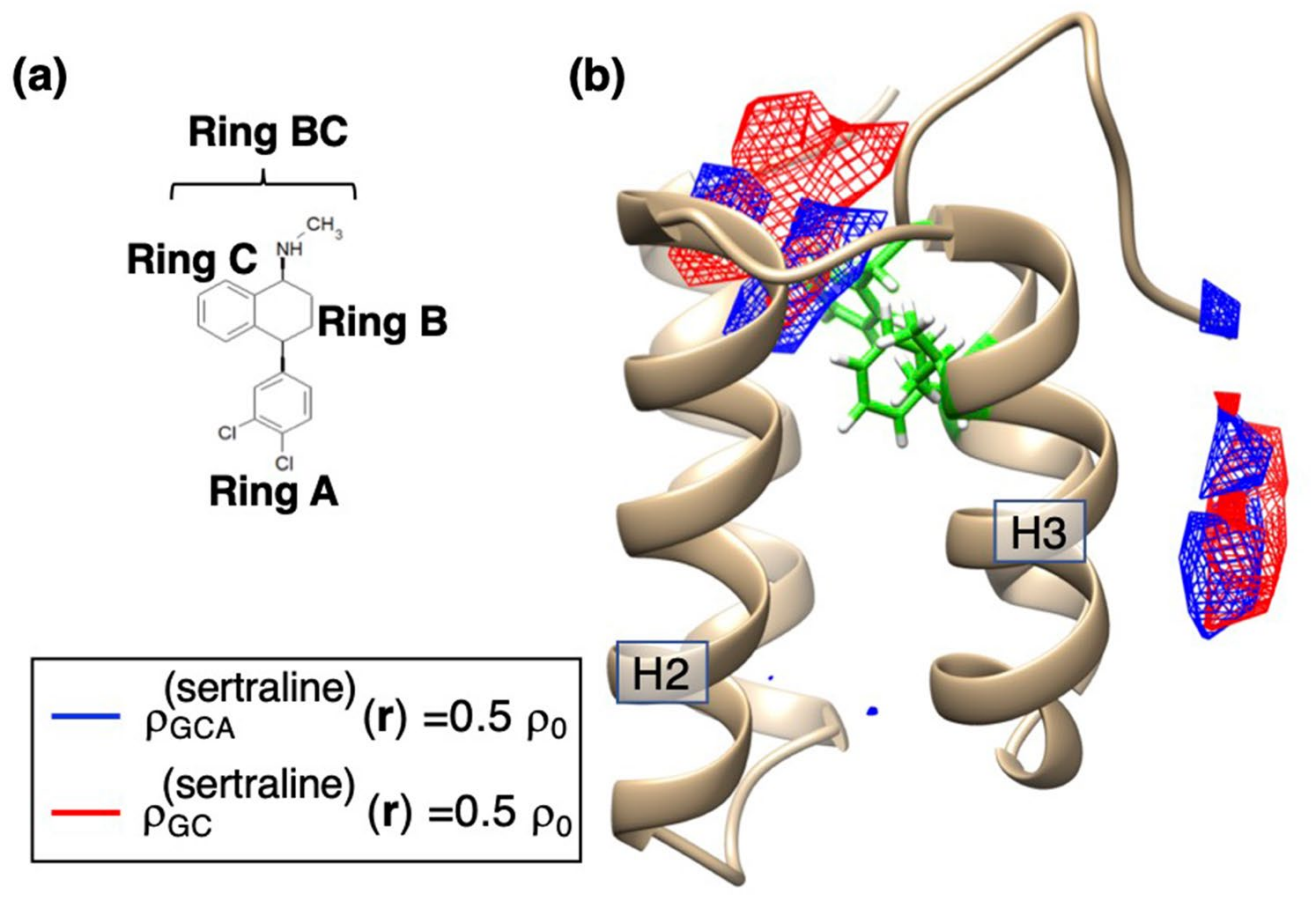
(**a**) Chemical structure of sertraline, where ring A and ring BC (rings A and B) are shown. (**b**) Spatial density $\rho_{CGA}^{{\left( {{\text{sertraline}}} \right)}} \left( {\varvec{r}} \right)$ (blue-colored contours) for the geometric center of Ring A of sertraline in the sertraline–mSin3B system, where the contour level is $\rho_{CGA}^{{\left( {{\text{sertraline}}} \right)}} \left( {\varvec{r}} \right) = 0.5\rho_{0}$ ($\rho_{0} = 0.001$). Red-colored contours are $\rho_{CG}^{{\left( {{\text{sertraline}}} \right)}} \left( {\varvec{r}} \right)$, which is spatial density of the geometric center of the entire sertraline. Labels H2 and H3 represent helices 2 and 3, respectively. Green-colored residues are Val 75, Phe 93, and Phe 96 of mSin3B. See Supplementary Section 4 for procedure to calculate $\rho_{CGA}^{{\left( {{\text{sertraline}}} \right)}} \left( {\varvec{r}} \right)$.

To investigate more the ligand–receptor interactions for the sertraline–mSin3B system, we calculated the spatial density $\rho_{GCA}^{{\left( {{\text{sertraline}}} \right)}} \left( {\varvec{r}} \right)$ of the geometrical center of Ring A of sertraline: See Fig. 4a for the position of Ring A of sertraline and Supplementary Eq. S4 for the procedure to calculate $\rho_{GCA}^{\left( s \right)} \left( {\varvec{r}} \right)$. Figure 4b illustrates $\rho_{GCA}^{{\left( {{\text{sertraline}}} \right)}} \left( {\varvec{r}} \right)$ and $\rho_{GC}^{{\left( {{\text{sertraline}}} \right)}} \left( {\varvec{r}} \right)$ with the contour level of $0.5\rho_{0}$. The contours in the mSin3B’s cleft correspond those for Cluster A in Fig. 2a. Thus, conformations taken from the contours are constituents of the most probable binding mode of the sertraline–mSin3B system. Ring A occupies the deeper position of the cleft than the geometrical center of the entire sertraline did. Thus, Ring A was closer to Val 75, Phe 93, and Phe 96 of mSin3B. In the next section, we investigate the conformations in this most probable binding mode. The contours near the C-terminal tail of mSin3B does to Cluster B, which is the artificial cluster as discussed before.

### Orientation of ligands bound to mSin3B

**Figure 5:**
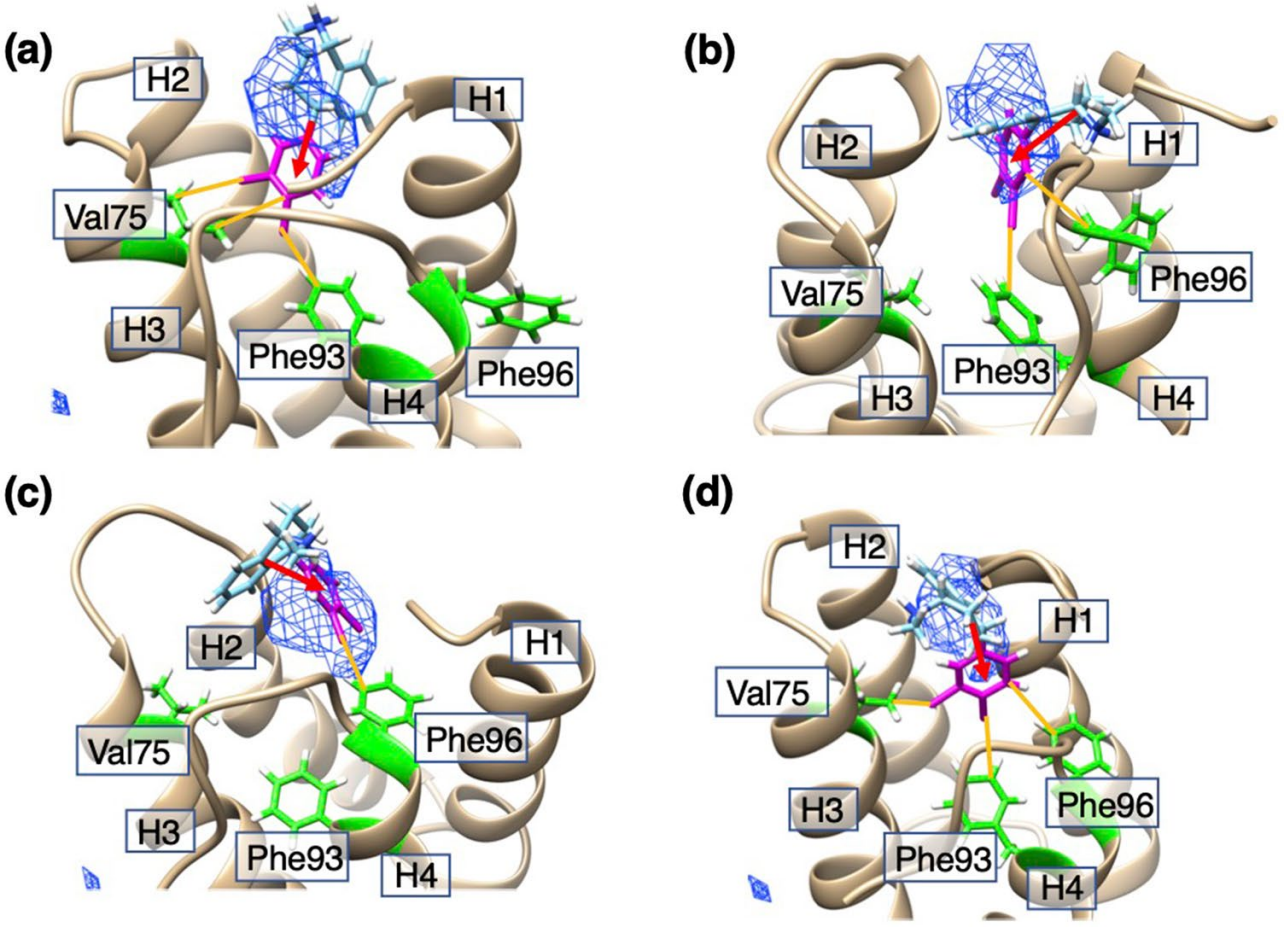
(**a**)–(**d**) Sertraline–mSin3B complexes picked randomly from the high-density cluster (Cluster A) of $\rho_{CG}^{{\left( {{\text{sertraline}}} \right)}} \left( {\varvec{r}} \right) > 0.5\rho_{0}$ ($\rho_{0} = 0.001$), which are shown by blue-colored contours. Magenta-colored portion is Ring A of sertraline. Red-colored arrows are those pointing from the Ring-BC geometrical center to that of Ring A (see Fig. 6a for positions of Ring A and BC). Green-colored residues are Val 75, Phe 93, and Phe 96 of mSin3B. Ocher-colored lines represent contacts between sertraline and the three residues. Labels H1,…, H4 are helices 1–4 of mSin3B (PAH1 domain).

The above analysis shows that the most stable/probable complex is not assigned to a single complex structure but to an ensemble of complex structures, which construct a high-density cluster. For the sertraline–mSin3B system, the most probable complex structures are those originated from Cluster A (Fig. 2a). Figure 5 demonstrates some conformations picked randomly from Cluster A. This figure also displays Val 75, Phe 93, and Phe 96 of mSin3B. Here we judged that Ring A was contacting to these residues when $r_{R}^{\left( s \right)}$ < 4.0 Å was satisfied. Remember that $r_{R}^{\left( s \right)}$ was defined in the previous subsection. In Fig. 5a Ring A contacted to Val 75 and Phe 93, and in Fig. 5b Ring A did to Phe 93 and Phe 96. Ring A contacted to Phe 96 in Fig. 5c, and Ring A did to all of the three residues in Fig. 5d. Examining a number of snapshots, we concluded that ligand–receptor contacts shown in Fig. 3a were contributed mainly by Ring A.

**Figure 6:**
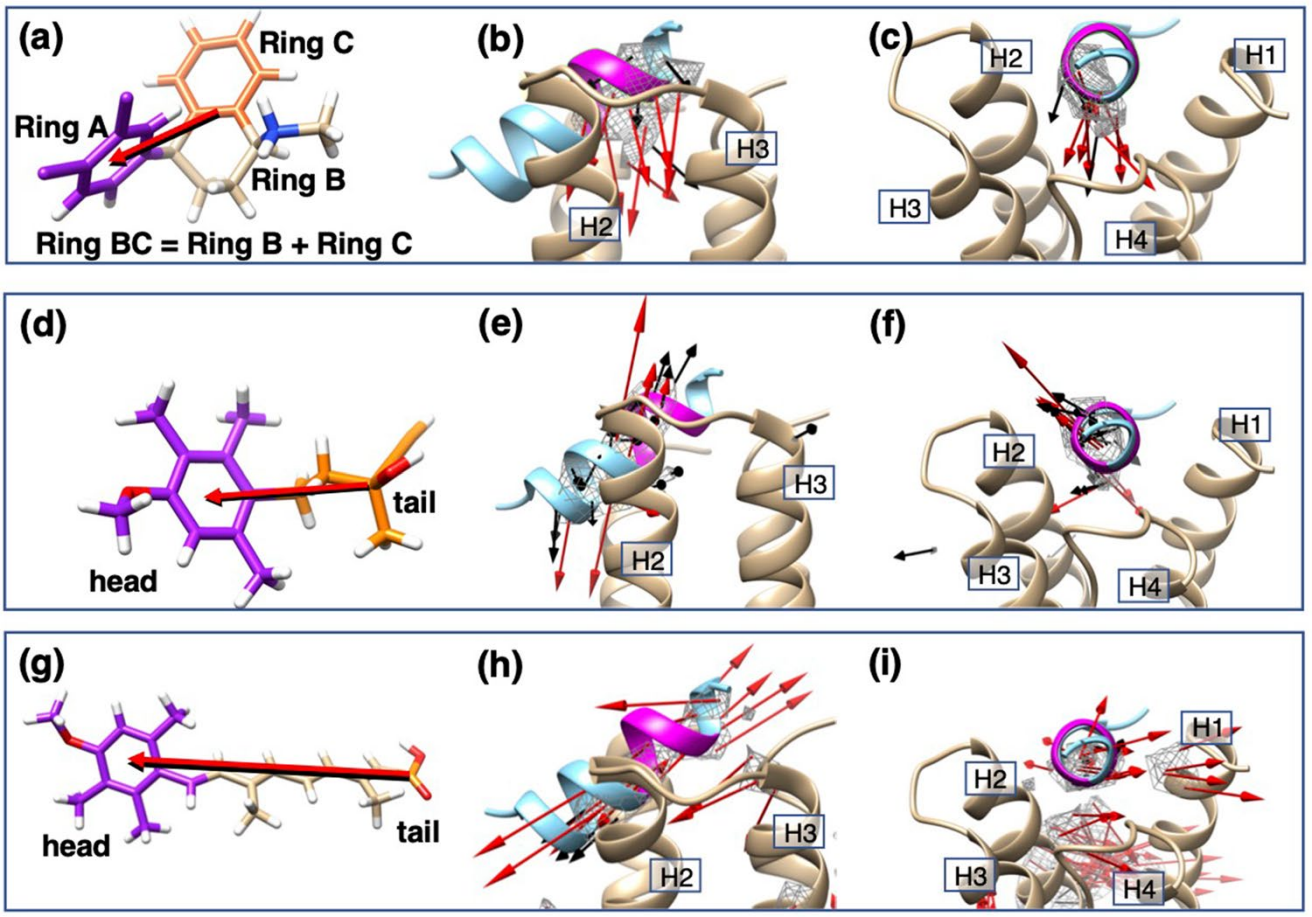
(**a**) A snapshot of sertraline, where three rings, Ring A, B, and C are defined and Rings B and C are unified as Ring BC. Ligand’s orientation (red-colored arrow) is defined by an arrow pointing from the geometric center of Ring BC to that of Ring A. Panels (**b**) and (**c**) illustrate spatial patterns of $\left\langle {{\varvec{e}}^{{\left( {{\text{sertraline}}} \right)}} \left( {\varvec{r}} \right)} \right\rangle$ in the cleft of mSin3B viewed from two different directions. The vectors $\left\langle {{\varvec{e}}^{{\left( {{\text{sertraline}}} \right)}} \left( {\varvec{r}} \right)} \right\rangle$ are assigned to high-density regions of $\rho_{CG}^{{\left( {{\text{sertraline}}} \right)}} \left( {\varvec{r}} \right) > 0.5\rho_{0}$ ($\rho_{0} = 0.001$) presented by gray contours. Red and black vectors are those with $\left| {\left\langle {{\varvec{e}}^{{\left( {{\text{sertraline}}} \right)}} \left( {\varvec{r}} \right)} \right\rangle } \right| \ge 0.5$ and $\left| {\left\langle {{\varvec{e}}^{{\left( {{\text{sertraline}}} \right)}} \left( {\varvec{r}} \right)} \right\rangle } \right| < 0.5$, respectively. This figure also displays REST/NRSF bound to mSin3B (PDB ID: 2CZY), and the magenta-colored segment is the LIML sequence of REST/NRSF. Note that REST/NRSF is not involved in the current simulation. Labels H1,…, H4 are helices 1,…, 4 of mSin3B (PAH1 domain). (**d**) A snapshot of YN3, where “head” and “tail” are colored by purple and orange, respectively. Red-colored arrow is the YN3’s orientation pointing from the geometric center of tail to that of head. Panels (**e**) and (**f**) illustrate spatial patterns of $\left\langle {{\varvec{e}}^{{\left( {{\text{YN}}3} \right)}} \left( {\varvec{r}} \right)} \right\rangle$. See captions for panels (**b**) and (**c**) for method to draw $\left\langle {{\varvec{e}}^{{\left( {{\text{YN}}3} \right)}} \left( {\varvec{r}} \right)} \right\rangle$ from two different directions. (**g**) A snapshot of acitretin, where “head” and “tail” are colored by purple and orange, respectively. Red-colored arrow is the acitretin’s orientation pointing from the geometric center of tail to that of head. Panels (**h**) and (**i**) illustrate spatial patterns of $\left\langle {{\varvec{e}}^{{\left( {{\text{acitretin}}} \right)}} \left( {\varvec{r}} \right)} \right\rangle$ from two different directions. See captions for panels (**b**) and (**c**) for method to draw $\left\langle {{\varvec{e}}^{{\left( {{\text{acitretin}}} \right)}} \left( {\varvec{r}} \right)} \right\rangle$.

Red-colored arrows in Fig. 5 shows the molecular orientations of sertraline has a tendency: Ring A was inserted into the binding cleft of mSin3B and Ring BC was left behind. To analyze more this tendency, we defined the molecular orientation of a snapshot for the three ligands: Fig. 6a, d, and g illustrate the ligand’s orientation for sertraline, YN3, and acitretin, respectively. Here, to investigate statistically the ligand’s orientation, we introduced a unit vector ${\varvec{e}}_{i}^{\left( s \right)}$ (*s* = sertraline, YN3, or acitretin) parallel to the ligand’s orientation for snapshot $i$, and calculated average, $\left\langle {{\varvec{e}}^{\left( s \right)} \left( {\varvec{r}} \right)} \right\rangle$, of the unit vectors over snapshots whose centers of mass were detected in each cube centered at ${\varvec{r}}$. The detailed procedure for averaging is presented in Supplementary Section 7.

Figure 6b and c demonstrate the spatial patterns of $\left\langle {{\varvec{e}}^{{\left( {{\text{sertraline}}} \right)}} \left( {\varvec{r}} \right)} \right\rangle$ for the sertraline–mSin3B system viewed from two different directions. This figure also indicates that Ring A tends to be inserted in the cleft of mSin3B, which is consistent to Fig. 5.

We also calculated $\left\langle {{\varvec{e}}^{{\left( {{\text{YN}}3} \right)}} \left( {\varvec{r}} \right)} \right\rangle$ for the YN3–mSin3B (Fig. 6e and f) and acitretin–mSin3B (Fig. 6h and i) systems. Figure 6h and i show that the acitretin’s orientation tends to be parallel or anti-parallel to the helical cylinder of REST/NRSF in the REST/NRSF-mSin3B complex. We presume that these acitretin’s orientations have an advantage to fit the whole acitretin’s framework to the binding cleft of mSin3B. To stabilize one of the parallel or anti-parallel orientations of acitretin, an additional inter-molecular interaction is required, which works differently between the two orientations. We consider that there is no such interaction to stabilize effectively one of the two orientations for the acitretin–mSin3B system.

On the other hand, the YN3’s orientations (Fig. 6e and f) were not aligned to the helical cylinder of REST/NRSF but tilting from the helical cylinder. We presume that this tilting of YN3 is because YN3 is smaller than acitretin: YN3 may be responding to undulations of mSin3B’s molecular surface in the cleft. This point is discussed later again.

### Flexibility of ligands’ framework

Figure 5 demonstrates the structural variety of the sertraline’s framework in the most probable complex state (Cluster A). To quantify the flexibility of the ligand’s framework, we calculated the distance distribution function, DDF, for a distance between two atoms set in each ligand. The procedure to calculate DDF for a distance is given in Supplementary Section 6.

**Figure 7:**
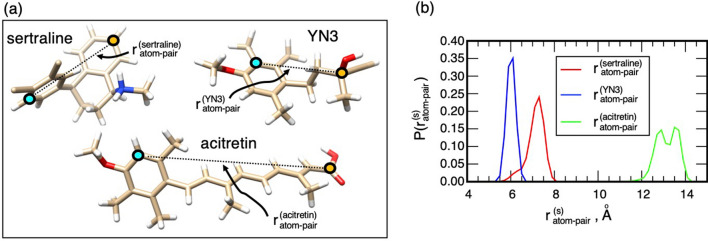
(**a**) Structures of the three compounds sertraline, YN3, and acitretin. For each compound, inter-atomic distance $r_{atom - pair}^{\left( s \right)}$ (*s* = sertraline, YN3, or acitretin) is defined between cyan- to orange-colored atoms. A cyan-colored atom, which is involved in a ring for each ligand, is not set on the ring rotation axis to detect the ring-rotational motions. (**b**) Distance distribution functions (DDFs), $P\left( {r_{atom - pair}^{\left( s \right)} } \right)$, for the three distances. See Supplementary Section 6 for procedure to calculate DDF.

Here, we picked the cyan- and orange-colored atoms in Fig. 7a from the framework of each ligand, and calculated the distance between the two atoms: $r_{atom - pair}^{\left( s \right)}$ (*s* = sertraline, YN3, or acitretin). The resultant DDFs $P\left( {r_{atom - pair}^{\left( s \right)} } \right)$ are displayed in Fig. 7b. The average of the distance $\left\langle {r_{atom - pair}^{\left( s \right)} } \right\rangle$ and its standard deviation $SD\left( {r_{atom - pair}^{\left( s \right)} } \right)$ (i.e., amount of fluctuations around $\left\langle {r_{atom - pair}^{\left( s \right)} } \right\rangle$) were 7.12 Å and 0.37 Å for sertraline, 6.01 Å and 0.14 Å for YN3, and 13.15 and 0.47 Å for acitretin. The largest SD was from acitretin. Therefore, one may consider that the acitretin’s framework is most flexible. However, acitretin is not the most flexible. Consider two bar-shaped objects (Object A and B) made of the same material, and suppose that the length of the bar of Object A is longer than that of Object B. Then, the following relation will be obtained: $SD\left( {{\text{object}}\;{\text{A}}} \right) > SD\left( {{\text{object}}\;{\text{B}}} \right)$. However, the flexibility of the two objects should be exactly the same because they are made of the same material. Then, we define the amount of fluctuations per unit length by $sd^{\left( s \right)} = SD\left( {r_{atom - pair}^{\left( s \right)} } \right){/}\left\langle {r_{atom - pair}^{\left( s \right)} } \right\rangle$, which is better to assess the molecular flexibility. The resultant $sd^{\left( s \right)}$ was $5.20 \times 10^{ - 2}$ for sertraline, $2.33 \times 10^{ - 2}$ for YN3, and $3.57 \times 10^{ - 2}$ for acitretin. From these values, the framework of sertraline is the most flexible, acitretin has a considerably stiffer framework than sertraline does, and YN3 is stiffest. The ligand’s flexibility may be related to the ligand’s molecular orientation as discussed later.

## Discussion

**Figure 8:**
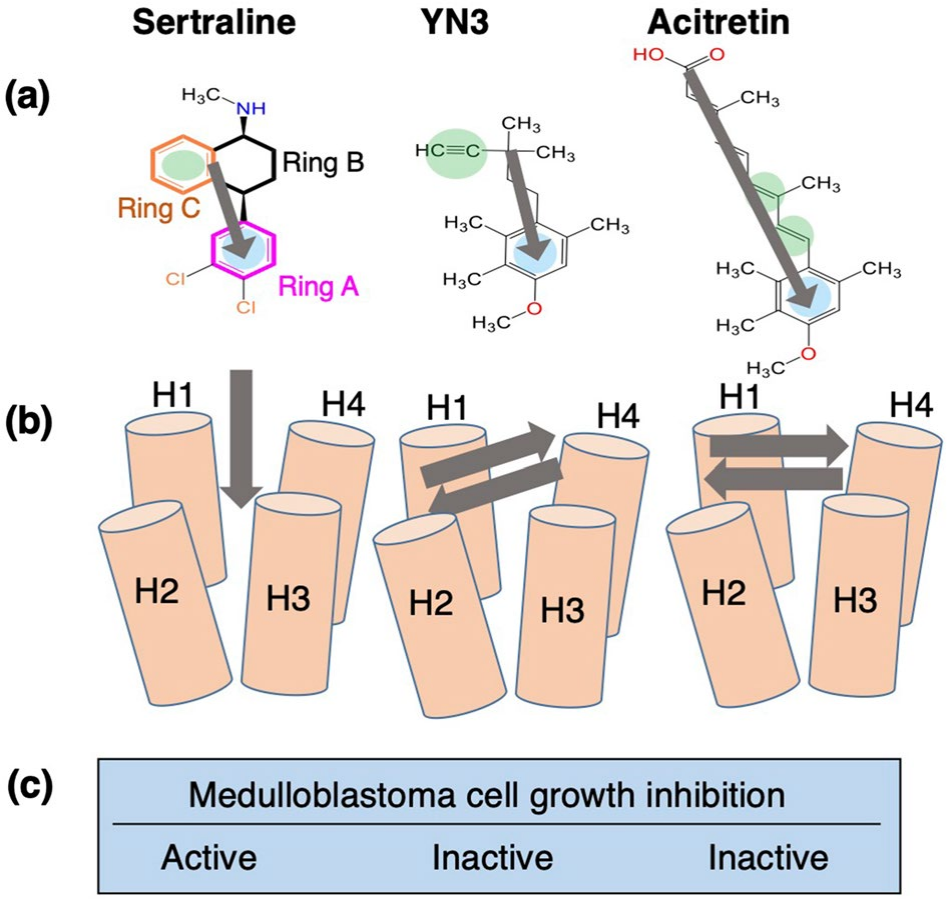
(**a**) Chemical structures of three compounds, and their orientations (gray arrows). Rings A, B and C of sertraline are depicted in magenta, black, and orange, respectively. Blue circles represent aromatic rings that are expected to interact with the hydrophobic cleft of mSin3B from conventional structure–activity relationship (SAR). Green circles represent $\pi$-electron rich regions. (**b**) Orientations of the compounds in the bound forms with mSin3B, resulted from the present MD simulation study. (**c**) Presence/absence of medulloblastoma cell-growth inhibition activities for the compounds.

Starting from the initial conformations where the ligands were distant from the cleft of mSin3B (Supplementary Figure S5), only sertraline reproduced a similar intermolecular hydrophobic core with that observed in the REST/NRSF-mSin3B complex (PDB ID: 2CZY): Ring A of sertraline, which is hydrophobic, bound deeply to the cleft of mSin3B with contacting to the hydrophobic sidechains sited in the cleft (Fig. 5). The binding scheme for the sertraline–mSin3B complex is summarized in the left column of Fig. 8. The hydrophobic core formed between Ring A of sertraline and the cleft of mSin3B was similar with that formed in the NRSF/REST–mSin3B complex. We presume that the sertraline–mSin3B complex formation competes with the REST/NRSF-mSin3B complex formation, and that this competition leads to the medulloblastoma cell-growth inhibition activity of sertraline.

It is interesting to compare the currently obtained complex structures (i.e., structures in Cluster A) with a modeled structure^31^, which was obtained from a docking software HADDOCK^54^. Although HADDOCK does not take the statistical-mechanical factors into the modeling, the receptor and ligand are modeled to bind to each other so as to satisfy experimental data, the chemical shift perturbation data^31^. Supplementary Figure S11 displays the HADDOCK complex structure using atomic coordinates presented by Kurita et al. Interestingly, Ring A sertraline in the HADDOCK model contacted to Phe 93 and Phe 96 (the ocher-colored lines in the figure), and then, Ring A was oriented somewhat toward the inside of the mSin3B’s cleft (the red-colored arrow in the figure). Remember that these structural features were found in our computed complex structures in Cluster A (Figs. 5 and 6a). We emphasize that the current simulation did not use the experimental data in computation.

Now, we discuss the interactions of acitretin with mSin3B. Acitretin has a low spatial density in the mSin3B’s cleft (Fig. 2c), which weakens the inhibitory activity partly. Furthermore, the framework of acitretin tends to be parallel or anti-parallel to the mSin3B’s cleft (Fig. 6c and the right panel of Fig. 8b). Remember that acitretin can be regarded as a long and stiff rod as shown in the above section. The parallel or anti-parallel molecular orientation is advantageous to be fit to the cleft. If acitretin has a flexible framework, acitretin may insert a portion into the hydrophobic cleft by bending the framework.

Next, we discuss the interactions of YN3 with mSin3B. Although YN3 had a high spatial density around the mSin3B’s cleft, YN3 did not sink deeply in the cleft and the hydrophobic core was not formed (Fig. 2c). This result is natural because YN3 can be regarded as a stiff rod. As discussed for acitretin above, the stiff rod fits well to the cleft taking the parallel or anti-parallel orientation. On the other hand, we presume that the slight tilt of $\left\langle {{\varvec{e}}^{{\left( {YN3} \right)}} \left( {\varvec{r}} \right)} \right\rangle$ to the helix cylinder of NRSF/REST in the NRSF/REST–mSin3B complex (Fig. 6b and the middle panel of Fig. 8b) is resulted from the small size of YN3. I.e., YN3 can adopt to the jaggedness of the inside of the cleft. If YN3 has more flexibility, YN3 may exert the inhibitory activity by inserting a molecular portion into the cleft with varying the molecular conformation. On the other hand, the added flexibility may induce binding of YN3 to the other surfaces of mSin3B than the cleft. These competitive effects of the molecular flexibility cannot be assessed only from the chemical structure of the compound.

We also note another chemical property of YN3 and acitretin, which is disadvantageous for interacting with hydrophobic cleft of mSin3B: Both rings in these compounds have an oxygen atom (Supplementary Figure S2b and c), which can interact to hydrophilic residues in mSin3B or water molecules electrostatically or by hydrogen bonding.

The conventional structure–activity relationship (SAR) study has been based on the chemical structures and target-binding activity data. Figure 8a shows one of the possible alignments of the three compounds based on their chemical structures. Each compound has an aromatic ring corresponding to Ring A and each aromatic ring has side chains in the para position. While sertraline has Cl atoms on Ring A, YN3 and acitretin have methoxy groups whose volumes are close to that of the Cl atom. Instead of Ring C of sertraline, YN3 and acitretin have triple and double bonds, respectively. These bonds are $\pi$-electron rich, and can form CH–$\pi$ interaction as same as aromatic rings. However, such SAR analysis could not explain presence and absence of the medulloblastoma cell-growth inhibition activities of their compounds (Fig. 8c). The present MD simulation study predicted the molecular orientations of the compounds (Fig. 8b) and the presence/absence of the hydrophobic core in the cleft of mSin3B (Fig. 2). Importantly, only sertraline could reproduce the binding mode in the NRSF/REST–mSin3B complex. We emphasize that the current simulation method, the GA-guided mD-vcMD simulation, produces a thermodynamically acceptable ensemble consisting of various conformations (bound and unbound conformations), and importantly a thermodynamic weight is assigned to each snapshot in the ensemble.

The preceding study^31^ classified 52 compounds into two pharmacophores, A and B, based on their chemical structures (see Fig. 2 of Ref.^31^), where sertraline belongs to Pharmacophore A and the YN3 to Pharmacophore B. Because acitretin has a structural similarity with YN3 apparently, acitretin belongs to Pharmacophore B. Based on Figs. 6 and 8b, the compounds belonging to Pharmacophore A have the parallel or anti-parallel orientation, and the compound belonging to Pharmacophore B has a perpendicular orientation. Therefore, the current MD procedure is useful if it is used with the pharmacophore analysis.

## Conclusions

**Figure 9:**
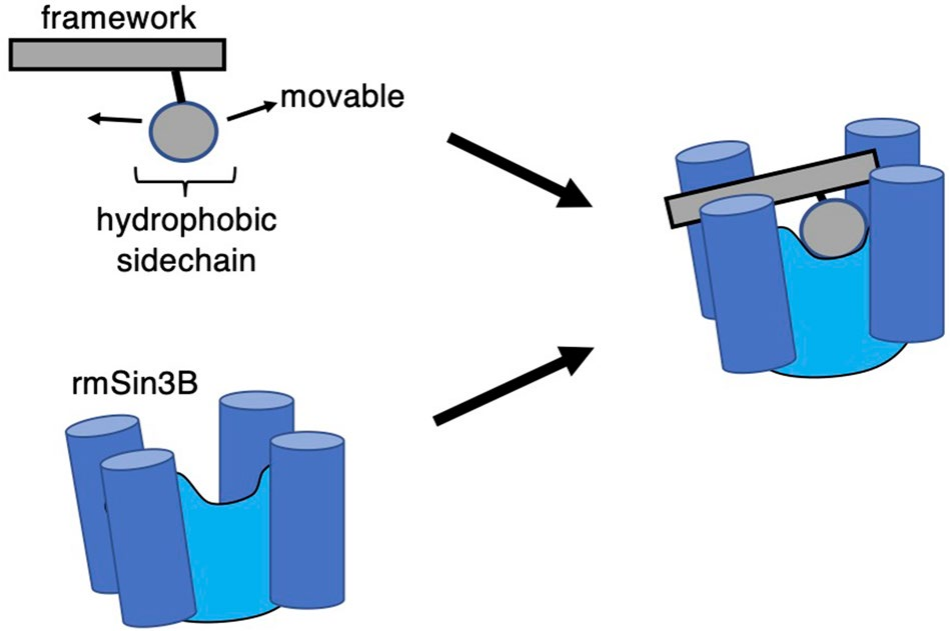
Scheme of a compound that binds to the cleft of mSin3B.

Binding of the compounds to mSin3B (PAH1 domain) was investigated by the GA-guided mD-VcMD simulation. This method produced useful quantities such as the spatial density of the ligand around the receptor (Fig. 2), the intermolecular contact patterns (Fig. 3), the propensity of molecular orientation (Figs. 5 and 6), and the ligand flexibility (Fig. 7). From these analyses, we showed that only sertraline produces a similar inter-molecular binding mode observed in the REST/NRSF-mSin3B complex. Figure 9 is a schematic drawing to design an inhibitor. Given a framework of the compound, by adding a hydrophobic sidechain to the framework, the hydrophobic core is formed between the sidechain and the hydrophobic cleft of mSin3B. The flexibility of the compound’s framework may increase the binding affinity, although a long and stiff framework may decrease the binding affinity. In general, it is difficult to specify the effect of the modification to the biological activity (i.e., inhibitory activity in the present study) only from the compound’s chemical structure. In contrast, the GA-guided mD-VcMD is useful to identify the effect with analyzing the thermally equilibrated conformational ensemble.

Because REST/NRSF is an intrinsically disordered segment that can bind to multiple proteins^3,4^ as mentioned in “Introduction”, section the current study is an example to design a compound that can inhibit binding of an intrinsically disorder segment to a protein receptor.

The 3D models have been submitted to the Biological Structure Model Archive (BSM-Arc) of the PDBj under BSM-ID BSM00020 (https://bsma.pdbj.org/entry/20), which are freely available^55^.

## Supplementary Information

Supplementary Information.
