# Supplementary material for: Difference of binding modes among three ligands to a receptor mSin3B corresponding to their inhibitory activities
Source: Sci Rep. 2021 Mar 17;11:6178. doi: 10.1038/s41598-021-85612-9 (PMC7971087; doi:10.1038/s41598-021-85612-9)
Supplement: Supplementary file 1 — Supplementary Information. [file 41598_2021_85612_MOESM1_ESM.pdf]

Supplementary Information

Difference of binding modes among three ligands to a receptor mSin3B corresponding to their inhibitory activities

Tomonori Hayami[1], Narutoshi Kamiya[2], Kota Kasahara[3], Takeshi Kawabata[1], Jun-ichi Kurita[4], Yoshifumi Fukunishi[5], Yoshifumi Nishimura[4,6], Haruki Nakamura[1], Junichi Higo[1,2,*]

[1] Institute for Protein Research, Osaka University, 3-2 Yamada-oka, Suita, Osaka 565-0871, Japan

[2] Graduate School of Simulation Studies, University of Hyogo, 7-1-28 Minatojima Minamimachi, Chuo-ku, Kobe, Hyogo 650-0047, Japan

[3] College of Life Sciences, Ritsumeikan University, 1-1-1 Noji-higashi, Kusatsu, Shiga 525-8577, Japan

[4] Graduate School of Medical Life Science, Yokohama City University, 1-7-29 Suehiro-cho, Tsurumi-ku, Yokohama, 230-0045, Japan

[5] Cellular and Molecular Biotechnology Research Institute, National Institute of Advanced Industrial Science and Technology (AIST), 2-3-26, Aomi, Koto-ku, Tokyo, 135-0064, Japan

[6] Graduate School of Integrated Sciences for Life, Hiroshima University, 1-4-4 Kagamiyama, Higashi-Hiroshima 739-8258, Japan

[*]Correspondence and requests for materials should be addressed to J.H. (e-mail: higo@protein.osaka-u.ac.jp) and K.K. (e-mail: ktkshr@fc.ritsumei.ac.jp)

Supplementary Figure S1.

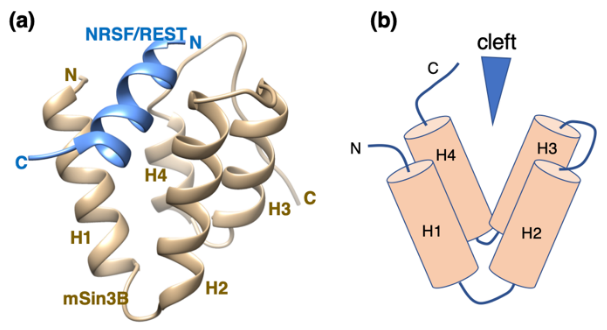

Figure S1: (a) Complex structure of PAH1 domain and NRSF/REST. In this paper, the PAH1 domain of mSin3B is denoted simply as "mSin3B". Four helices, composing mSin3B, are denoted as $H1$–$H4$. The "C" and "N" are respectively the N- and C-termini for each chain. Shown structure is a snapshot from a sampling simulation[1]. (b) Schematic drawing of the mSin3B structure. The binding cleft of mSin3B is shown by a triangle.

Supplementary Figure S2.

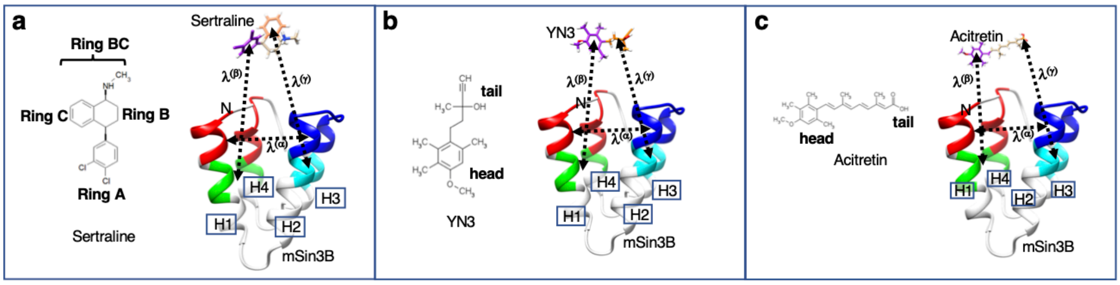

Figure S2: (a) (left) Chemical structure of sertraline where three rings are named as "Ring A", "Ring B", "Ring C" in this study. Rings B and C are combined and called "Ring BC" simply. (right) Three reaction coordinates (RCs), $\lambda^{(\alpha)}$, $\lambda^{(\beta)}$, and $\lambda^{(\gamma)}$, are introduced in the sertraline–mSin3B system. (b) (left) Chemical structure of YN3. The ring is named as "head", and the opposite side of the ring as "tail" in this study. (right) Three reaction coordinates, $\lambda^{(\alpha)}$, $\lambda^{(\beta)}$, and $\lambda^{(\gamma)}$, are introduced in the YN3–mSin3B system. (c) (left) Chemical structure of acitretin. The ring is named as "head", and the opposite side of the ring as "tail" in this study. (right) Three reaction coordinates, $\lambda^{(\alpha)}$, $\lambda^{(\beta)}$, and $\lambda^{(\gamma)}$, are introduced in the acitretin–mSin3B system. In each panel, four helices of mSin3B are indicated by "H1", "H2", "H3", and "H4" from the N- to C-terminal. Label "N" shows the position of the N-terminal of mSin3B. $\lambda^{(\alpha)}$ is defined by the distance between the center of mass of red-colored segments of mSin3B and that of blue-colored segment of mSin3B. $\lambda^{(\beta)}$ is defined by the distance between the center of mass of green-colored segments of mSin3B and that of purple-colored part of compound. $\lambda^{(\gamma)}$ is defined by the distance between the center of mass of cyan-colored segments of mSin3B and that of orange-colored part of compound.

Supplementary Section 1. Definition of a reaction coordinate (RC).

Consider two atom groups G_h^A and G_h^B $(h = \alpha, \beta, \gamma, \ldots)$ in a molecular system. The reaction coordinate (RC) $\lambda^{(h)}$ is defined by the distance between centers of mass of G_h^A and G_h^B (Supplementary Figure S3). Superscripts A and B indicate simply that two atom groups are pairing to define $\lambda^{(h)}$, and then, one can exchange the superscripts as: $G_h^A \rightarrow G_h^B$ and $G_h^B \rightarrow G_h^A$ without changing the value of $\lambda^{(h)}$.

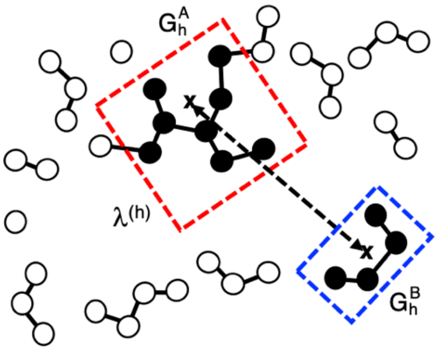

Figure S3: Two atom groups, G_h^A and G_h^B, are indicated by red-colored and blue-colored rectangles, respectively. Atoms in G_h^A and G_h^B are presented by small black filled circles. Center of mass of each atom group is presented by a cross. The distance between the two centers of mass is $\lambda^{(h)}$ (broken-line with arrows).

Supplementary Figure S4.

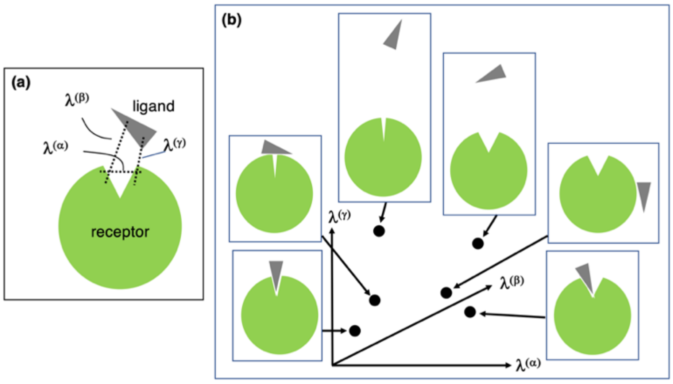

Figure S4: (a) Scheme of three RCs, $\lambda^{(h)}$ $(h = \alpha, \beta, \gamma)$, introduced for the current ligand–receptor system: Variation of $\lambda^{(\alpha)}$ controls the cleft opening/closing of the receptor. Variations of $\lambda^{(\beta)}$ and $\lambda^{(\gamma)}$ control ligand approaching/departing from receptor, and relative orientation of the ligand with respect to the receptor. (b) Three-dimensional RC space constructed by $\lambda^{(\alpha)}$, $\lambda^{(\beta)}$, and $\lambda^{(\gamma)}$, where phase point (filled circle) moves according to the system's conformational motion.

Supplementary Table S1. Atom groups to define three RCs.

RC	Atom Group[a)] G_h^A	G_h^B
$\lambda^{(\alpha)}$	residues 33–38, 93–99[b)] of mSin3B	residues 63–75 of mSin3B
$\lambda^{(\beta)}$	residues 40–43, 90–92 of mSin3B	purple-colored portion in ligand in Fig. S1-S3 of SI
$\lambda^{(\gamma)}$	residues 60–62, 77–80 of mSin3B	orange-colored portion in ligand in Fig. S1-S3 of SI

[a)] See Section 1 of Supplementary Information for definition of atom groups G_h^A and G_h^B $(h = \alpha, \beta, \gamma)$. Superscripts A and B are assigned to indicate the atom groups G_h^A and G_h^B, which are pairing to define $\lambda^{(h)}$.

[b] "residues n_1–n_2" involves all atoms in residues n_1–n_2 in mSin3B. See also Fig. S1-S3 for positions of the atom groups in mSin3B.

Supplementary Table S2. Parameters[a] for RC-space division

h	$n_{vs}(h)$[a]	$[\lambda_1^{(h)}]_{min}$[b]	$[\lambda_{n_{vs}(h)}^{(h)}]_{max}$[b]	$\Delta\lambda^{(h)}$[c]
α	7	10.0 Å	18.0 Å	2.0 Å
β	19	0.0 Å	25.0 Å	2.5 Å
γ	19	0.0 Å	25.0 Å	2.5 Å

[a] Number of virtual states for each reaction coordinate $\lambda^{(h)}$.

[b] Variable range for $\lambda^{(h)}$ is $\left[[\lambda_1^{(h)}]_{min}, [\lambda_{n_{vs}(h)}^{(h)}]_{max}\right]$.

[c] Zone width for each reaction coordinate $\lambda^{(h)}$.

Supplementary Section 2. Restraints applied to the C-terminal tail of mSin3B.

We introduced distance-restraint energy, E_{res}, between a part of PAH1 domain of mSin3B and its C-terminal tail to prevent the C-terminal tail from being inserted into the binding cleft of PAH1 domain. The function form is:

$$E_{res} = \begin{cases} 0.5\sum_{i,j}\left[r_{i,j} - (r_{i,j}^0 - r_{low})\right]^2 & (\text{for } r_{i,j} \le r_{i,j}^0 - r_{low}) \\ 0 & (\text{for } r_{i,j}^0 - r_{low} < r_{i,j} < r_{i,j}^0 + r_{up}) \\ 0.5\sum_{i,j}\left[r_{i,j} - (r_{i,j}^0 + r_{up})\right]^2 & (\text{for } r_{i,j} \ge r_{i,j}^0 + r_{up}) \end{cases} \quad \text{(S1)}$$

where $r_{i,j}$ and $r_{i,j}^0$ are the Cα atomic distance between residues i and j in a simulation snapshot and the reference complex structure (the NMR structure of the NRSF/REST-mSin3B complex; PDB ID: 2CZY), respectively, and r_{low} and r_{up} specify tolerances set to 2.0 Å. Thus, no restraint ($E_{rst} = 0$) is applied to the atom-pair distance $r_{i,j}$ when $r_{i,j}^0 - r_{low} < r_{i,j} < r_{i,j}^0 + r_{up}$. This distance restraint was applied to three Cα atomic distance pairs between Gly 92 and Asp 104, between Phe 93 and Ile 105, and between Asn 94 and Arg 106.

Supplementary Figure S5.

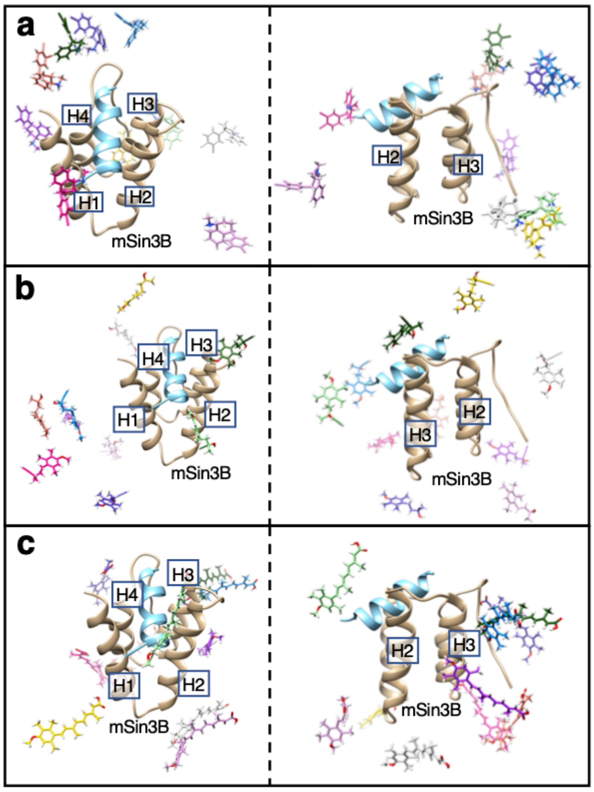

Figure S5: (a) Some initial conformations of GA-guided mD-VcMD for the sertraline-mSin3B system viewed from two different directions. Solvent is omitted. Shown mSin3B structure is the NMR structure (PDB ID: 2CZY). This figure also displays the NRSF/REST fragment (cyan-colored model) binding to mSin3B in the NMR structure, while NRSF/REST does not exist in the GA-guided mD-VcMD simulation. Labels H1,…, H4 are helices 1–4 of mSin3B (PAH1 domain). (b) Some initial conformations for the YN3-mSin3B system viewed from two different directions. (c) Some initial conformations for the acitretin-mSin3B system viewed from different directions.

Supplementary Section 3. Parameters used.

The atom groups adopted for the current study to define the three RCs are given in Table S1 of SI. Three RCs are illustrated in Figure S2 of SI.GA-guided mD-VcMD consists of iterative simulations, through which the conformational ensemble converges on an equilibrated one, $Q_{cano}(\boldsymbol{\lambda})$ $(\boldsymbol{\lambda} = \lambda^{(\alpha)}, \lambda^{(\beta)}, \lambda^{(\gamma)})$, at a simulation temperature (300 K) in the 3D-RC space. A thermodynamic weight is assigned to each of stored snapshots using $Q_{cano}(\boldsymbol{\lambda})$ [2,3]. This means that a thermally equilibrated conformational ensemble (canonical ensemble) is obtained in the allowed RC space.

Table S2 lists actual values of parameters which control the simulations: $n_{vs}(h)$, $[\lambda_1^{(h)}]_{min}$, $[\lambda_{n_{vs}(h)}^{(h)}]_{max}$, and $\Delta\lambda^{(h)}$. Table S3 lists the zones: $\{[\lambda_k^{(h)}]_{min}, [\lambda_k^{(h)}]_{max};\ k = 1, \dots, n_{vs}(h)\}$. An interzone transition was attempted once every $20\,ps$ $(1 \times 10^4$ steps of simulation). See Ref. 2 for meaning of parameters.

Supplementary TableS3. Setting of zones

Zone No. [a)]	zones [b)]					
k	$[\lambda_k^{(\alpha)}]_{min}$	$[\lambda_k^{(\alpha)}]_{max}$ [c)]	$[\lambda_k^{(\beta)}]_{min}$	$[\lambda_k^{(\beta)}]_{max}$	$[\lambda_k^{(\gamma)}]_{min}$	$[\lambda_k^{(\gamma)}]_{max}$ [d)]

1	10.0	12.0	0.00	2.50
2	11.0	13.0	1.25	3.75
3	12.0	14.0	2.50	5.00
4	13.0	15.0	3.75	6.25
5	14.0	16.0	5.00	7.50
6	15.0	17.0	6.25	8.75
7	16.0	18.0	7.50	10.00
8			8.75	11.25
9			10.00	12.50
10			11.25	13.75
11			12.50	15.00
12			13.75	16.25
13			15.00	17.50
14			16.25	18.75
15			17.50	20.00
16			18.75	21.25
17			20.00	22.50
18			21.25	23.75
19			22.50	25.00

[a] Number of zones n_{vs} is 7 for $\lambda^{(\alpha)}$ and 19 for $\lambda^{(\beta)}$ and $\lambda^{(\gamma)}$.

[b] Unit of zones is Å.

[c] $[\lambda_k^{(\alpha)}]_{min}$ $[\lambda_k^{(\alpha)}]_{max}$ are not assigned for $k \geq 8$ because number of virtual states is 7: $n_{vs}(\alpha) = 7$. See Table S2.

[d] Parameters for $\lambda^{(\gamma)}$ are not shown because they are exactly the same as those for $\lambda^{(\alpha)}$.

Supplementary Section 4. Spatial density of ligand's quantity.

The GA-guided mD-VcMD assigns a thermodynamic weight (statistical weight at equilibrium) to each sampled snapshot[2]. Here we present a method to calculate spatial distribution of the center of mass of the ligands around the receptor mSin3B. First, we divide the 3D real space into cubes, whose volume ΔV is 2 Å $\times$ 2 Å $\times$ 2 Å. The cube position is specified by its center $\boldsymbol{r} = [x, y, z]$. Next, we calculate the geometrical center (GC) of the ligand for each snapshot, and assign the snapshot to a cube that involves the GC. Then, we assign all of the snapshots to cubes. Last, we calculate the spatial density $\rho_{GC}(\boldsymbol{r})$ of the geometrical center at the cube $\boldsymbol{r}$ as:

$$\rho_{GC}^{(s)}(\boldsymbol{r}) = \sum_i w_i \delta_{GC}(\boldsymbol{r}; i), \tag{S2}$$

where w_i is the thermodynamic weight assigned to snapshot i, and the superscript s specifies the ligand: $s =$ sertraline-mSin3B, YN3-mSin3B, or acitretin-mSin3B. The function $\delta_{GC}(\boldsymbol{r}; i)$ is a delta function defined:

$$\delta_{GC}(\boldsymbol{r}; i) = \begin{cases} 1 & (\text{if GC of snapshot } i \text{ is in cube } \boldsymbol{r}) \\ 0 & (\text{else}) \end{cases}. \tag{S3}$$

Equation S2 of Supplementary Information is the thermodynamic weight assigned to the cube $\boldsymbol{r}$. We normalized $\{w_i\}$ as $\sum_i w_i = 1$ for each system in advance.

Another spatial density function is calculated with the same manner. For instance, a spatial density $\rho_{GCA}^{(s)}(\boldsymbol{r})$ for the geometrical center of Ring A of sertraline (GCA) is calculated as follows:

$$\rho_{GCA}^{(s)}(\boldsymbol{r}) = \sum_i w_i \delta_{GCA}(\boldsymbol{r}; i), \tag{S4}$$

where

$$\delta_{GCA}(\boldsymbol{r}; i) = \begin{cases} 1 & (\text{if GCA of snapshot } i \text{ is in cube } \boldsymbol{r}) \\ 0 & (\text{else}) \end{cases}. \tag{S5}$$

The superscript s is set to sertraline-mSin3B.

Supplementary Section 5. Convergence of distribution.

Figure 1 of the main text demonstrates the conformational distribution $Q_{cano}(\lambda^{(\alpha)}, \lambda^{(\beta)}, \lambda^{(\gamma)})$ in the 3D RC space for the three systems computed from the GA-guided mD-VcMD. Here, we access the convergence of $Q_{cano}(\lambda^{(\alpha)}, \lambda^{(\beta)}, \lambda^{(\gamma)})$ with proceeding the iteration (i.e., the ordinal number of iteration). The assessment is done with $E_{local}(\lambda^{(\alpha)}, \lambda^{(\beta)}, \lambda^{(\gamma)})^{-1}$ that is a function defined locally at each position in 3D RC space. This function was introduced in our paper[2] to assess the accuracy of $Q_{cano}(\lambda^{(\alpha)}, \lambda^{(\beta)}, \lambda^{(\gamma)})$, and used actually to check the simulation quality in another paper[3]. The larger the function ${E_{local}}^{-1}$ at a position $[\lambda^{(\alpha)}, \lambda^{(\beta)}, \lambda^{(\gamma)}]$, the better the accuracy of Q_{cano} at the position. According to Ref. 3, we judged that a 3D-RC region with ${E_{local}}^{-1} \geq 4.0$ has an appropriate accuracy.

Supplementary Figures S6–S8 demonstrate the regions with $E_{local}(\lambda^{(\alpha)}, \lambda^{(\beta)}, \lambda^{(\gamma)})^{-1} \geq 4.0$, which increased with proceeding the iteration of simulation. Those regions covered the regions of $Q_{cano}\left(\lambda^{(\alpha)}, \lambda^{(\beta)}, \lambda^{(\gamma)}\right) \geq 0.01$ relatively well in the last iteration for each system (the 14-th, 13-th, and 27 the iterations for the sertraline-mSin3B, YN3-mSin3B, and acitretin-mSin3B systems, respectively).

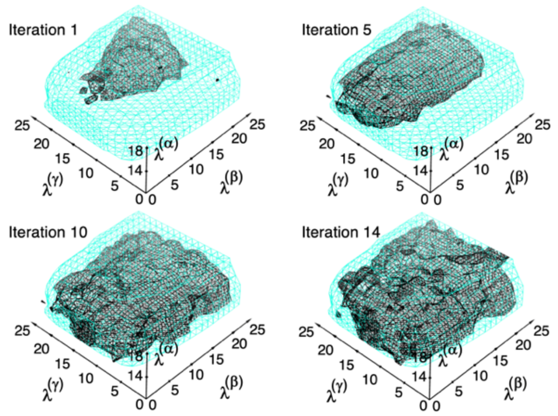

Figure S6: Convergence of distribution $Q_{cano}(\lambda^{(\alpha)}, \lambda^{(\beta)}, \lambda^{(\gamma)})$ with proceeding iterative simulation for the sertraline-mSin3B system. Accuracy of $Q_{cano}(\lambda^{(\alpha)}, \lambda^{(\beta)}, \lambda^{(\gamma)})$ is assessed by objective function $E_{local}\left(\lambda^{(\alpha)}, \lambda^{(\beta)}, \lambda^{(\gamma)}\right)^{-1}$.[2] Cyan contours, i.e., the equidensity region of $Q_{cano}\left(\lambda^{(\alpha)}, \lambda^{(\beta)}, \lambda^{(\gamma)}\right) = 0.001$, are those used in Figure 1 of main text. Black contours are the iso-objective-function surfaces of $E_{local}\left(\lambda^{(\alpha)}, \lambda^{(\beta)}, \lambda^{(\gamma)}\right)^{-1} = 4$. The iteration No. of each panel is indicated near the panel.

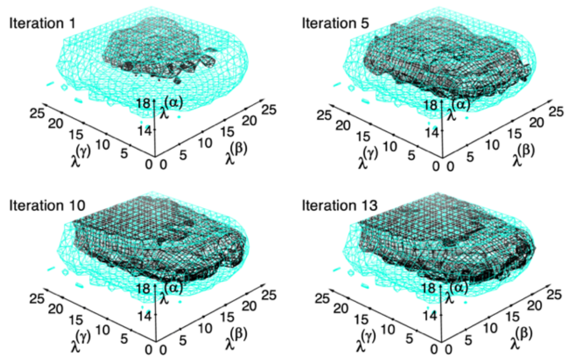

Figure S7: Convergence of distribution $Q_{cano}(\lambda^{(\alpha)}, \lambda^{(\beta)}, \lambda^{(\gamma)})$ with proceeding iterative simulation for the YN3-mSin3B system. See caption of Figure S6 for more information.

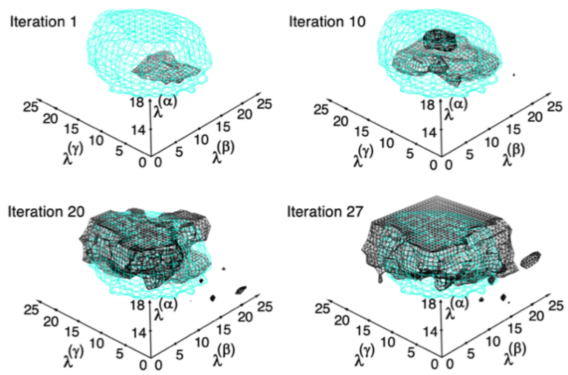

Figure S8: Convergence of distribution $Q_{cano}(\lambda^{(\alpha)}, \lambda^{(\beta)}, \lambda^{(\gamma)})$ with proceeding iterative simulation for the acitretin-mSin3B system. See caption of Figure S6 for more information.

Supplementary Figure S9.

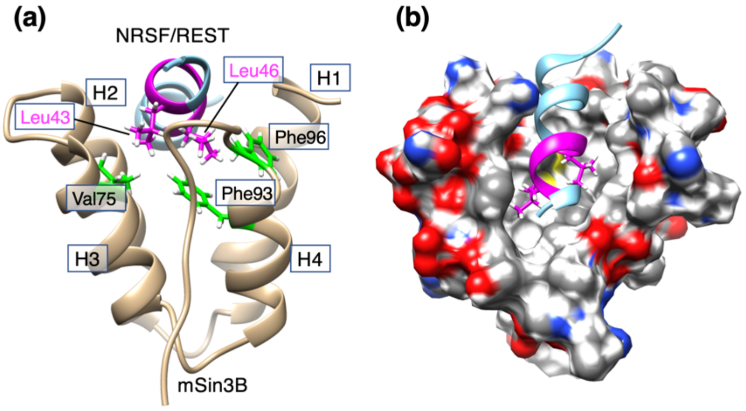

Figure S9: NRSF/REST–mSin3B complex structure determined by NMR (PDB ID: 2CZY). (a) The LIML sequence of NRSF/REST is shown by magenta-colored ribbon model, and two hydrophobic residues Leu 43 and Leu 46 (magenta-colored labels) of the LIML sequence are displayed explicitly. These two residues contact to three hydrophobic residues Val 75, Phe 93, and Phe 96 (green-colored residues with black labels) of mSin3B. Labels H1,…, H4 indicate helices 1,…, 4 of mSin3B (PAH1 domain), respectively. (b) mSin3B is presented by a surface model, where hydrophobic surface is represented by white color.

Supplementary Figure S10.

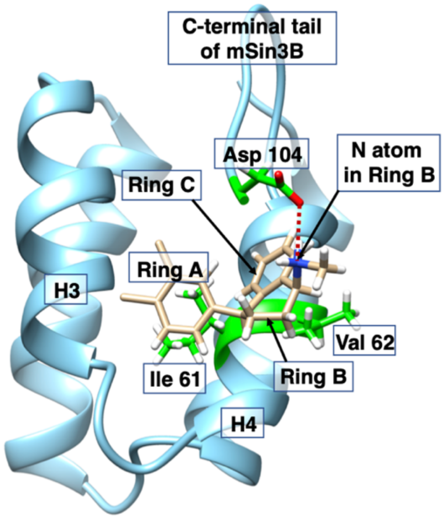

Figure S10: Conformation of sertraline taken from Cluster B. See Figure 2a of main text for definition of Cluster B. The nitrogen atom of Sertraline's Ring B interacts electrostatically to an oxygen atom of Asp 104 of the C-terminal tail of mSin3B, which is shown by brown-colored broken line. The distance between the two atom is 2.8 Å. Hydrophobic contacts are formed between sertraline's Ring A and Ile 61 of helix H4 of mSin3B as well as between sertraline's Ring C and Val 62.

Supplementary Section 6. Radial distribution function.

Defining a distance r between two objects in a system, we can calculate a distance distribution function (DDF) $P(r)$, where the probability of detecting r in a window $[r, r + dr]$ is $P(r)dr$. The normalization of DDF is defined as $\int_0^\infty P(r)dr = 1$. We use w_i (Supplementary section 4) to calculate the DDF. Then, the radial distribution function

(RDF) $p(r)$ is defined formally as: $p(r) = P(r)/4\pi r^2$. This function is normalized as $\int_0^\infty p(r) 4\pi r^2 dr = 1$.

In the present study, we calculate the minimum heavy-atomic distance $r_R^{(s)}$ between the ligand and one of three residues Val 75, Phe 93, and Phe 96 in the mSin3B cleft, where the subscript R of $r_R^{(s)}$ is the residue specifier ($R =$ Val 75, Phe 93, or Phe 96) and the superscript s is the system specifier defined in Eq. S2 of Supplementary Information. Then, we calculated three RDFs $p(r_R^{(s)})$ for each system.

Supplementary Section 7. Averaged molecular orientation in each cube.

Molecular orientation vectors for the compounds are defined by the red-colored vectors in Figures 6a, 6d, and 6g. Then, we defined a unit vector parallel to the molecular orientation vector for each snapshot: $\boldsymbol{e}_i^{(s)}$ where the subscript i is the snapshot specifier and superscript s is the system specifier. Then, the average of $\boldsymbol{e}_i^{(s)}$ at each cube was defined as:

$$< \boldsymbol{e}^{(s)}(\boldsymbol{r}) > = \frac{1}{\sum_i w_i \delta_{GC}(\boldsymbol{r}; i)} \sum_i \boldsymbol{e}_i^{(s)} w_i \delta_{GC}(\boldsymbol{r}; i). \quad \text{(S6)}$$

We calculated $< \boldsymbol{e}^{(s)}(\boldsymbol{r}) >$ in cubes whose densities were $\rho_{GC}^{(s)}(\boldsymbol{r}) \geq 0.5\rho_0$ for sertraline and YN3, and $\rho_{GC}^{(s)}(\boldsymbol{r}) \geq 0.1\rho_0$ for acitretin. We aim to investigate the spatial pattern of $< \boldsymbol{e}^{(s)}(\boldsymbol{r}) >$ in the high-density regions. Thus, we set the threshold for sertraline and YN3 was set to $0.5\rho_0$ referring to Figs. 2a and 2b. On the other hand, the regions of $0.5\rho_0$ did not appear around the cleft of mDin3B substantially for acitretin (Figure 2c). Thus, we decreased the threshold to $0.1\rho_0$ for acitretin.

Note that $|< \boldsymbol{e}^{(s)}(\boldsymbol{r}) >|$ represents a degree of ligand orientational ordering in each cube. $|< \boldsymbol{e}^{(s)}(\boldsymbol{r}) >|$ takes the maximum of 1 when all $\boldsymbol{e}_i^{(s)}$ have exactly the same orientations in the cube. If snapshots have uncorrelated orientations, then $|< \boldsymbol{e}^{(s)}(\boldsymbol{r}) >|$ becomes small.

Supplementary Figure S11.

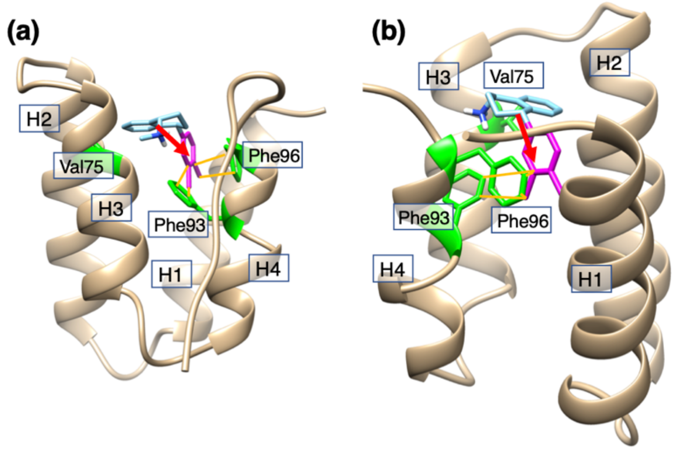

Figure S11: Sertraline-mSin3B complex viewed from two different orientations (a) and (b), which was modeled by Kurita et al.[4] using HADDOCK modeling[5]. The atomic coordinates were provided from Kurita et al. In the HADDOCK modeling, the receptor and ligand bind to each other so as to satisfy chemical shift perturbation data. The magenta-colored portion is Ring A of sertraline. Red-colored arrow is the ligand's orientation vector pointing from the Ring-BC geometrical center to that of Ring A. Green-colored residues are Val 75, Phe 93, and Phe 96 of mSin3B. Ocher-colored lines represent contacts between sertraline and the three residues. Labels H1,…, H4 are helices 1–4 of mSin3B (PAH1 domain).

Supplementary References.

1. Higo, J., Nishimura, Y. & Nakamura, H. A Free-energy landscape for coupled folding and binding of an intrinsically disordered protein in explicit solvent from detailed all-atom computations. *J. Am. Chem. Soc.*, **133**, 10448–10458 (2011).
2. Higo, J., Kusaka, A., Kasahara, K., Kamiya, N., Hayato, I., Qilin, X., Takahashi, T., Fukuda, I., Mori, M., Hata, Y. & Fukunishi, Y. GA-guided mD-VcMD: A genetic-algorithm-guided method for multi-dimensional virtual-system coupled molecular dynamics. *Biophysics and Physicobiology* **17**, 161–176 (2020).
3. Higo, J., Kawabata, T., Kusaka, A., Kasahara, K., Kamiya, N., Fukuda, I., Mori, K., Hata, Y., Fukunishi, Y. & Nakamura, H. Molecular interaction mechanism of a 14-3-3 protein with a phosphorylated peptide elucidated by enhanced conformational sampling. *J. Chem. Inf. Model.* **60**, 4867–4880 (2020).
4. Kurita, J., Hirao, Y., Nakano, H., Fukunishi, Y. & Nishimura, Y. Sertraline, chlorprothixene, and chlorpromazine characteristically interact with the REST-binding site of the corepressor mSin3, showing medulloblastoma cell growth inhibitory activities. *Scientific Reports* **8**, 13763 (2018).
5. Dominguez, C., Boelens, R. & Bonvin, A. M. J. J. Haddock: a protein-protein docking approach based on biochemical or biophysical information. *J. Am. Chem. Soc.* **125**, 1731–1737 (2003).
